# Antiviral activity of phenanthrenes from the medicinal plant *Bletilla striata* against influenza A virus

**DOI:** 10.1186/s12906-017-1780-6

**Published:** 2017-05-22

**Authors:** Ya Shi, Bing Zhang, Yiyu Lu, Chaodong Qian, Yan Feng, Liwei Fang, Zhishan Ding, Dongqing Cheng

**Affiliations:** 1 College of Medical Technology, Zhejiang Chinese Medical University, Hangzhou, Zhejiang 310053 China; 2 Zhejiang Provice Center For Disease Control and Prevention, Hangzhou, Zhejiang 310051 China; 3 College of Life Science, Zhejiang Chinese Medical University, Hangzhou, Zhejiang 310053 China; 4 Department of Biological Sciences, University of Wisconsin-Milwaukee, Milwaukee, 53211 USA

**Keywords:** *Bletilla striata*, Embryonated, Influenza A virus, Phenanthrene

## Abstract

**Background:**

Influenza represents a serious public health concern. The emergence of resistance to anti-influenza drugs underlines the need to develop new drugs. This study aimed to evaluate the anti-influenza viral activity and possible mechanisms of 12 phenanthrenes from the medicinal plant *Bletilla striata* (*Orchidaceae* family)*.*

**Methods:**

Twelve phenanthrenes were isolated and identified from *B. striata*. Influenza virus A/Sydney/5/97 (H3N2) propagated in embryonated chicken eggs was used. Phenanthrenes mixed with the virus were incubated at 37 °C for 1 h and then inoculated into 9-day-old embryonated chicken eggs via the allantoic route to survey the antiviral activity in vivo. A (3-(4,5-dimethylthiazol-2-yl)-5-(3-carboxymethoxyphenyl)-2-(4-sulfophenyl)-2H–tetrazolium) (MTS)-based assay was performed to evaluate the reduction of cytopathic effect induced by H3N2 on Madin-Darby canine kidney (MDCK) cells. The hemagglutination inhibition assay was used to study the blockage of virus receptors by the phenanthrenes, and the neuraminidase (NA) inhibition assay to evaluate the effects of the release of virus. The synthesis of influenza viral matrix protein mRNA in response to compound treatment was measured by real-time polymerase chain reaction.

**Results:**

This study showed that phenanthrenes 1, 2, 3, 4, 6, 9, 10, 11, and 12 significantly inhibited the viruses in vivo, with inhibition rates of 20.7, 79.3, 17.2, 34.5, 34.5, 34.5, 44.8, 75.9, and 34.5%, respectively. In MDCK models, the phenanthrenes did not show significant antiviral activity when administered as pretreatment, while phenanthrenes 2, 3, 4, 6, 7 10, and 11 exhibited inhibitory activities as simultaneous treatment with 50% inhibition concentration (IC_50_) ranging from 14.6 ± 2.4 to 43.3 ± 5.3 μM. The IC_50_ ranged from 18.4 ± 3.1 to 42.3 ± 3.9 μM in the post-treatment assays. Compounds 1, 3, 4, 6, 10, and 11 exhibited an inhibitory effect on NA; and compounds 2, 3, 4 6, 7, 10, and 11 resulted in the reduced transcription of virus matrix protein mRNA. However, no compound could inhibit hemagglutination by the influenza virus.

**Conclusion:**

Phenanthrenes from *B. striata* had strong anti-influenza viral activity in both embryonated eggs and MDCK models, and diphenanthrenes seemed to have stronger inhibition activity compared with monophenanthrenes.

**Electronic supplementary material:**

The online version of this article (doi:10.1186/s12906-017-1780-6) contains supplementary material, which is available to authorized users.

## Background

Influenza viruses are responsible for seasonal epidemics and occasional pandemics, which cause significant morbidity and mortality. The vaccines need to be reformulated almost every year owing to antigenic drift, and the timely production of pandemic vaccines remains problematic because of the limitations of current technology [1–4]. Antiviral drugs play a significant role in controlling the spread of the disease, but the emergence of drug-resistant viral strains has been reported occasionally [5, 6], which has become a serious public health concern globally. Therefore, safe and effective new antiviral drugs need to be developed urgently to combat viral infections for either therapeutic or prophylactic purposes.

Two surface glycoproteins are present on the envelope of the influenza virus: hemagglutinin (HA) and neuraminidase (NA). The life cycle of the influenza virus begins with viral HA binding to sialic acid (SA) receptors on the host cell surface, followed by internalization of the virus by receptor-mediated endocytosis [7, 8]. Subsequently, endosomal acidification alters the conformation of HA and leads to the fusion of the host and viral membranes, which allows the release of the viral nucleoproteins (NPs) into the cytoplasm. In the nucleus of the infected cells, the viral RNAs are transcribed into mRNAs and replicated. Finally, the newly synthesized viral ribonucleoproteins (vRNPs) are exported into the cytoplasm, and after packaging, mature virions are released from the cell surface depending on the cleaving of SA receptors by sialidase. Therefore, HA plays a key role in initiating viral infection by binding to SA-containing receptors on the host cells, thus mediating the subsequent viral entry and membrane fusion [9–11]. The NA cleaves the specific linkage of the SA receptor, resulting in the release of the newly formed virions from the infected cells [12, 13]. Oseltamivir, marketed under the trade name Tamiflu, was the first orally administered commercially developed NA inhibitor. It was discovered using shikimic acid as a starting point for synthesis. Shikimic acid was originally available as an extract of Chinese star anise. Oseltamivir is on the World Health Organization’s list of essential medicines, a list of the most important medications needed in a basic health system. However, it was found that a substantial number of patients might become oseltamivir-resistant as a result of oseltamivir use and that oseltamivir resistance might be significantly associated with pneumonia [14, 15].

The tuber of *Bletilla striata* (*Orchidaceae* family) is a well-known traditional Chinese herb (known as Baiji in Chinese). The Chinese Pharmacopoeia states that *B. striata* supports hemostasis and detumescence and promotes recovery. Phytochemical research revealed that *Bletilla* contains polysaccharides, bibenzyl, phenanthrene, dihydrophenanthrene, flavonoids, and phenolic compounds [16–19]. In addition, it was reported to have antimicrobial [20], antioxidant [20], and anti-inflammatory activities [21]. The chemical components of *B. striata* and its 2,2-diphenyl-1-picrylhydrazyl (DPPH) radical-scavenging, ferric-reducing antioxidant, and tyrosinase-inhibitory activities were studied [22]. Furthermore, six biphenanthrenes were separated, and their antibacterial activities were reported [23]. In the present study, the anti-influenza viral activity of 12 phenanthrenes was investigated, and the antiviral mechanisms, such as inhibition of the activity of NA or HA, were explored.

## Methods

### Preparation of the compounds

The rhizomes of *B. striata* (*Orchidaceae* family) were collected from Tuankou Town, Zhejiang Province, People’s Republic of China, and authenticated by Prof. ZS Ding (one of the authors). A voucher specimen was deposited at the Zhejiang Chinese Medical University with specimen number BS-2012-I. The organic extract of *B. striata* was prepared via maceration in 95% ethanol under reflux four times (each time, 100 min). After removal of the solvent under reduced pressure, the residual was suspended in 1 L of H_2_O and partitioned with EtOAc (1 L × 4) to yield an EtOAc-soluble fraction. The fraction was purified on a silica gel column eluted with a gradient CHCl3-MeOH solvent system (100:1, 75:1, 50:1, 25:1, 5:1, and 1:1) and then purified using a high-performance liquid chromatography (HPLC) system (260 nm, 1 mL/min) on a Venusil XBP C18 column (Bonna-Agela, USA) (250 × 10 mm^2^, 5 μM) eluted with a gradient mixture of acetonitrile in water with 0.1% formic acid [22, 23]. The structures were determined using an extensive spectroscopic analysis including UV, LC-MS, 1H–NMR, and 13C–NMR.

### Virus propagation and titration

The influenza virus strain A/Sydney/5/97 (H3N2) used in this study was obtained from Zhejiang Provincial Center for Disease Control and Prevention, propagated in the allantoic cavity of 9- to 11-day-old chicken eggs at 34 °C, and harvested 48 h after inoculation as the pooled allantoic fluid [24, 25]. After a brief centrifugation (3000 rpm at 4 °C for 20 min) and hemagglutination titer measured by a hemagglutination test (WHO, 2002), the virulence of the virus was determined by a 50% egg infective dose (EID_50_) analysis in chicken eggs [24, 26] and a 50% tissue culture infective dose (TCID_50_) analysis in Madin-Darby canine kidney (MDCK) epithelial cells [26, 27].

**Fig. 1:**
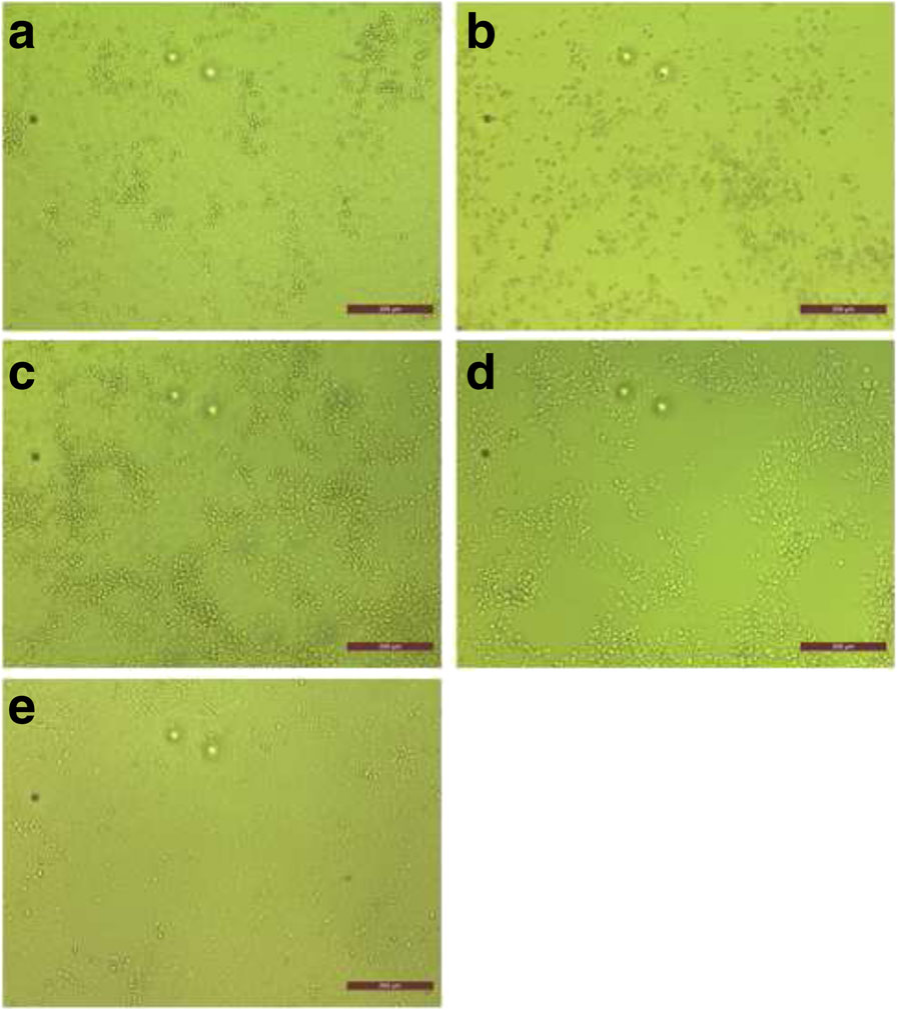
HPLC and LC–MS analyses of the compounds isolated from *Bletilla striata.* **a** HPLC fingerprint chromatogram of the 12 phenanthrenes isolated from *Bletilla striata.* **b** Total ion current chromatogram of the 12 phenanthrenes isolated from *Bletilla striata*, in the negative ion mode LC-MS. **c** Total ion current of chromatogram of the 12 phenanthrenes isolated from *Bletilla striata*, in the positive ion LC-MS

A series of tenfold dilutions of the virus were inoculated into the chick allantoic cavity (0.2 mL/embryo), each dilution with 10 embryos, at 34 °C for 48 h, and then harvested. A hemagglutination test was performed, and the positive rate of each dilution was recorded. EID_50_ was evaluated using the method of Reed and Muench [24, 26]. Approximately 5 × 10^4^ cells/well were seeded in 96-well microplates in minimum essential medium (MEM) with 10% fetal bovine serum (FBS) at 37 °C in a humidified 5% CO_2_ incubator. When the monolayer was confluent, the cells were washed twice with phosphate-buffered saline (PBS) and then infected with a series of tenfold virus dilutions (no-virus used as a control and each dilution with 10 replicates). The maintenance medium (MEM containing 100 U/mL penicillin G and 100 μg/mL streptomycin) was supplemented with 10 μg/mL L-1-(tosyl-amido-2-phenyl) ethyl chloromethyl ketone-treated trypsin (Sigma-Aldrich Company Ltd, UK) and then incubated at 37 °C with 5% CO_2_. The development of cytopathic effect (CPE) in the host cells was observed daily until no further CPE was observed and all no-virus control cells remained normal. CPE was recorded as five grades: ++++ (75–100% rounding of cells, increased refractility, or loss of adherence/detachment); +++ (50–74%); ++ (25–49%); + (1–24%); − (no morphologic changes in all the cells) (Fig. 1). The TCID_50_ was evaluated using the method of Reed and Muench [26, 27].

### Antiviral activity in embryonated eggs

Various concentrations of the compounds were prepared by diluting with PBS and mixed with equal volumes of influenza virus at 100 EID_50_. The compound-virus mixture was incubated for 1 h at 37 °C. The 9-day-old swabbed eggs were punched and inoculated with the compound-virus mixture via the allantoic route. PBS and the virus without treatment were used as controls. The positive control was oseltamivir phosphate. Triplicate tests were performed for each compound against the virus. The eggs were incubated at 34 °C for 48 h. The allantoic fluid was harvested, and the virus titer was measured using a hemagglutination test. The logarithmic transformation (log 2 calculations) was done for HA titers of the influenza virus, and the inhibitory activity was calculated. Inhibition (%) = (X - Y)/X × 100%, where X is the log 2 HA titer of the viral control, and Y is the log 2 HA titer of the tested samples [24, 25].

### Cytotoxicity to MDCK cells

An MTS-based assay was performed to evaluate the cytotoxic effects of the test compounds on MDCK cells. Approximately 5 × 10^4^ cells/well were seeded in 96-well microplates in MEM with 10% FBS at 37 °C in a humidified 5% CO_2_ incubator. When the monolayer was confluent, the cells were washed twice with PBS and then treated with compounds diluted in serum-free MEM. After incubation at 37 °C for 48 h, 20 μL of MTS was added to each well, and the cells were incubated for 2 h. The absorbance at 490 nm was determined using a microplate reader. The cell viability was calculated using the following formula: cell viability (%) = (drug treatment – background)/(control – background) × 100%. The half-maximal cytotoxic concentration (CC_50_) of the compounds was defined as the concentration that reduced the A_490_ of compound-treated cells to 50% of that of untreated cells [27, 28].

### Reduction of CPE in MDCK cells

Three tests were developed, and the non-cytotoxic concentration of each compound was determined using the cytotoxicity test. (1) Pretreatment: non-cytotoxic concentrations of the compounds were added to the cells, incubated for 18 h, and then removed. The cells were washed and infected with the influenza A/Sydney/5/97 (H3N2) at 100 TCID_50_ for 1 h. The virus was removed, and the medium was replaced with serum-free MEM as the maintenance medium. (2) Simultaneous treatment: non-cytotoxic concentration of the compounds and the virus at 100 TCID_50_ were added to the cell monolayers simultaneously at 37 °C for 1 h, and then supplemented with the maintenance medium. (3) Post-treatment: MDCK cells were infected with the virus at 37 °C for 1 h. Then the test compounds were added for 1 h and then supplemented with the maintenance medium. Cells without any treatment were used as not-infected control. PBS instead of the compounds was used as the infected control and oseltamivir phosphate as the positive control. Each test was performed in triplicate. All cultures were incubated for 72 h at 37 °C under 5% CO_2_ atmosphere until the infected control showed complete CPE: almost 100% of the cells were affected and most of the cell sheet came off the wall of the culture well as observed under a light microscope [25]. Then, 20 μL of MTS was added to each well, and the cells were incubated for 2 h. The absorbance at 490 nm was determined using a microplate reader. CPE Inhibition (%) = (*A*_drug treatment_ – *A*_infected control_)/(*A*_not-infected control_ – *A*_infected control)_ × 100%. The 50% inhibition concentration (IC50) was calculated using the Reed-Muench analysis [26–28].

### Hemagglutination inhibition assay

The hemagglutination inhibition assay was used to study whether the compound could block the SA-containing receptors. The phenanthrenes from *B. striata* and oseltamivir phosphate were all tested Fresh guinea pig blood was collected at the Laboratory Animal Research Center of the Zhejiang Chinese Medical University, supplemented with 1.6% sodium citrate in sterile water. Then, red blood cells (RBC) were separated by centrifugation (800×*g* for 10 min at room temperature), washed three times with sterile PBS, and then suspended at a concentration of 1.5%. The least number of virus particles able to agglutinate the guinea pig RBC was determined in a serial virus dilution as four hemagglutination units (HAU). PBS instead of the compounds was used as the hemagglutinating positive control and no-virus RBC as the hemagglutinating negative control. Then, 50 μL of the compounds in serial twofold dilutions in PBS were mixed with 50 μL of 4 HAU influenza virus suspension, incubated for 1 h at 37 °C, mixed with an equal volume of 1.5% guinea pig RBC suspension for 1 h at room temperature, and then monitored for agglutination [10, 11].

### Neuraminidase inhibition assay

The neuraminidase inhibition assay was used to evaluate the effects of each compound on the neuraminidase activity of the influenza virus, according to the instructions of the Neuraminidase Inhibitors Screen Kit (Beyotime, China). Inhibition = [(*C*− *S*) / (*C* − *C*_0_)] × 100%, where *C* is the fluorescence of the control (enzyme, buffer, and substrate) after 30 min of incubation, and *C*_0_ is the fluorescence of the control (buffer and substrate). *S* is the fluorescence of the tested samples (enzyme, sample solution, and substrate) after incubation. Standard curves were made by plotting the percentage of fluorescence inhibition relative to the activity of virus controls against the log_2_ of concentrations. In addition, 10 μg/mL oseltamivir phosphate was used as the positive control. Then, 10 μL of NA in 70 μL of reaction buffer was mixed with equal volumes of twofold dilutions of the compounds. After a 2-min incubation at 37 °C, equal volumes (10 μL) of the substrate solution [2-(4-methylumbelliferyl)-α-D-*N-*acetylneuraminic acid sodium (4-MU-NANA)] were added, and the mixtures were further incubated for 30 min at 37 °C. The fluorescence intensity was measured using a fluorescence plate reader at an excitation wavelength of 322 nm and an emission wavelength of 450 nm. The 50% inhibitory concentration (IC_50_) was calculated by regression analysis with *r* ^2^ ≥ 0.9, representing the mean values of three independent experiments [13, 29].

### Evaluation of the expression levels of matrix protein mRNA of the influenza virus using quantitative real-time polymerase chain reaction (RT-PCR)

The expression level of matrix protein mRNA of the influenza virus was determined by reverse transcription and quantitative RT-PCR to evaluate the effects of each compound on virus replication. MDCK cells were grown to approximately 90% confluence in 24-well plates with 4 × 10^5^ cells per well at 37 °C under 5% CO_2_, infected with the influenza virus at 100 TCID_50_, and cultured in the presence of the compounds at a concentration of 8 μM. PBS and the virus without treatment were used as controls, and the positive control was oseltamivir phosphate. The medium was removed after 18 h. The cells were scraped off, washed twice with PBS, and collected by centrifugation (500×*g* for 3 min). Total RNA was isolated using an RNeasy Mini Kit (Qiagen, Germany) according to the manufacturer’s instructions. RT-PCR was performed using a One-Step PrimeScript RT-PCR Kit (TaKaRa Biotech, Dalian, China). The primer sequences used for quantitative real-time PCR of viral matrix protein mRNA were 5′-GAC CRA TCC TGT CAC CTC TGA C-3′ (sense) and 5′-AGG GCA TTY TGG ACA AAK CGT CTA-3′ (antisense) [30]. Glyceraldehyde 3-phosphate dehydrogenase (GAPDH) was used as an internal control of cellular RNAs, with primer sequences of 5′-CAA CGG ATT TGG CCG TAT TGG-3′ (sense) and reverse: 5′-TGA AGG GGT CAT TGA TGG CG-3′ (antisense) [11]. RT-PCR was conducted in a 25-μL reaction system: 2× One-Step RT-PCR Buffer III, 12.5 μL; TaKaRa Ex Taq HS (5 U/μL), 0.5 μL; PrimeScript RT Enzyme Mix II, 0.5 μL; PCR Forward Primer (20 μM) 0.6 μL; PCR Reverse Primer (20 μM), 0.6 μL; SYBR Green Dye, 1 μL; total RNA, 3 μL; and RNase-Free dH_2_O, 6.3 μL. The amplification conditions were as follows: 40 °C for 30 min, 95 °C for 2 min, 95 °C for 5 s, and 55 °C for 35 s (40 cycles). The data were analyzed using the mode for normalized expression (2^–ΔΔCt^) [31].

### Statistical analysis

Data was presented as mean ± standard deviation (SD). Comparisons for all pairs were performed by the Student *t* test using SPSS version 17.0 (SPSS, IL, USA). A *P* value of <0.05 was considered to be significant. The IC_50_ and CC_50_ values were calculated using GraphPad Prism (GraphPad Software Inc., San Diego, CA, USA).

## Results

**Fig. 2:**
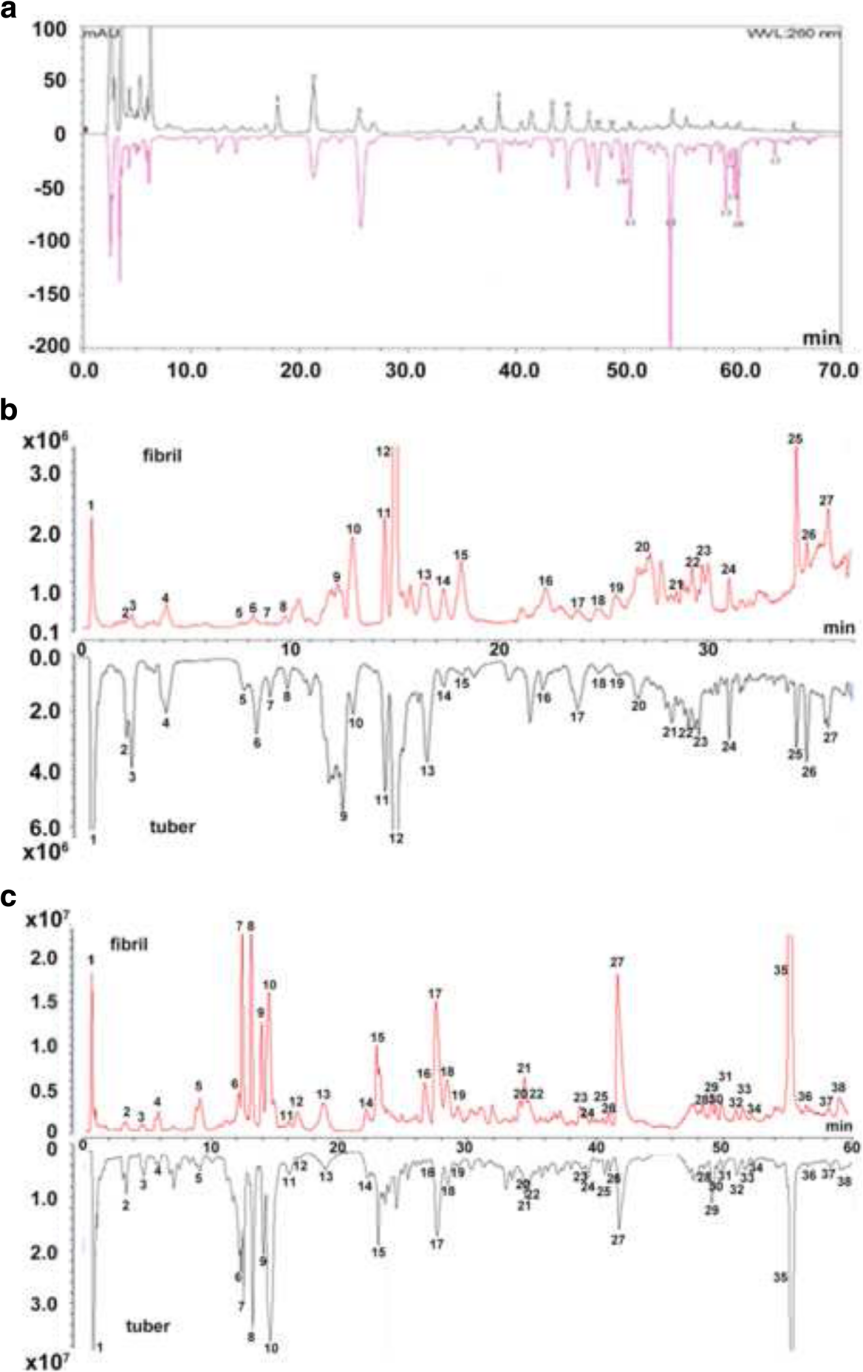
Influenza virus-induced cytopathic effect. The five panels present different degrees of cytopathic effect in Madin-Darby canine kidney epithelial cells. **a** +; **b** ++; **c** +++; **d** ++++; **e** Normal control

We have isolated 12 phenanthrenes from the medicinal plant *Bletilla striata*. The structures and names of the phenanthrenes were listed (Table 1); the chromatogram (Fig. 2) and spectra (Additional file 1), as well as the spectral data for all the compounds (Additional file 1) were also provided. The compounds 1, 9, 10 and 12 have been reported before, while the compounds 2, 3, 4, 5, 6, 7, 8 and 11 were new discoveries of our study.

**Table 1:** Chemical structures of phenanthrenes from the tuber of *Bletilla striata*

Structure		Name
1		2,7-dyhydroxyl-4-methoxy-9,10-dihydro-phenanthrene
2		2,2,7′-trihydroxy-4,4′,7-trimethoxy-9′,10′-dihydro-1,1′-diphenanthrene
3		2,2′,7′-trihydroxy-3′,4,5′,7-tetramethoxy-9′,10′-dihydro-1,1′-di-phenanthrene
4		4,4′,7,7′-tetrahydroxy-2,2′,8,8′-tetramethoxy-1,1′-di-phenanthrene
5		4,4′,7′-trihydroxy-2,2′,8,-trimethoxy-1,1′-di-phenanthrene
6		4,4′,7,7′-tetrahydroxy-2,2′-dimethoxy-1,1′-di-phenanthrene
7		4,4′,7-trihydroxy-2,2′,7′-trimethoxy-1,1′-di-phenanthrene
8		4,4′,7,7′-tetrahydroxy-2,2′,8-trimethoxy-1,1′-di-phenanthrene
9		4,5-dyhydroxyl-2-methoxy-9,10-dihydro-phenanthrene
10		2-hydroxyl-4,7-dimethoxyphenanthrene
11		2,2′–dyhydroxyl-4,4′,7,7′-9′,10′-dihydro-1,6′-di-phenanthrene
12		2,7-dyhydroxyl-4-methoxyphenanthrene

### Antiviral activity in embryonated eggs

The influenza virus A/Sydney/5/97 (H3N2) was propagated in 9-day-old chicken eggs; the dose was evaluated as 10^7.8^ EID_50_/mL. The suitable dilutions of all the compounds were set according to their solubility and toxicity. Then, 9-day-old swabbed eggs were inoculated with the compound-100 EID_50_ virus mixture via the allantoic route at 34 °C for 48 h. The virus titer of the allantoic fluid was measured by the hemagglutination test. The result showed that the *B. striata* compounds had significant antiviral activity against the influenza virus in the embryonated hen egg model. Compared with the control, nine compounds could significantly decrease the titers of viruses, with inhibition rates of 20.7% (compound 1), 79.3% (compound 2), 17.2% (compound 3), 34.5% (compound 4), 34.5% (compound 6), 34.5% (compound 9), 44.8% (compound 10), 75.9% (compound 11), and 34.5% (compound 12), at a concentration of 0.08 mmol/egg (Table 2). Compounds 2 and 11 were the best, although they seemed less efficient than oseltamivir, which showed a 100% inhibition rate at 0.01 mmol/egg. The results for other concentrations are presented (Table 2).

**Table 2:** Antiviral activity of the compounds from *Bletilla striata* in embryonated hen eggs

Compounds	Concentration (mmol/egg)	-log2 HA titer (mean ± SD)	Inhibition (%)
1	0.08	4.60 ± 0.55**	20.69
	0.16	2.20 ± 0.45**	62.07
	0.32	0.40 ± 0.55**	93.10
2	0.02	5.80 ± 0.84	0.00
	0.04	3.00 ± 0.71**	48.28
	0.08	1.20 ± 0.45**	79.31
3	0.02	6.20 ± 0.84	0.00
	0.04	5.40 ± 0.55	6.90
	0.08	4.80 ± 0.45**	17.24
4	0.04	6.00 ± 0.71	0.00
	0.08	3.80 ± 0.84**	34.48
	0.16	0.00 ± 0.00**	100.00
5	0.02	5.80 ± 0.84	0.00
	0.04	5.40 ± 0.55	6.90
	0.08	5.60 ± 0.55	3.45
6	0.04	5.80 ± 0.84	0.00
	0.08	3.80 ± 0.84**	34.48
	0.16	0.00 ± 0.00**	100.00
7	0.02	6.80 ± 0.45	0.00
	0.04	6.60 ± 0.55	0.00
	0.08	6.40 ± 0.55	0.00
8	0.04	5.40 ± 0.55	6.90
	0.08	2.20 ± 0.45**	62.07
	0.16	0.40 ± 0.55**	93.10
9	0.04	6.00 ± 0.71	0.00
	0.08	3.80 ± 0.84**	34.48
	0.16	0.00 ± 0.00**	100.00
10	0.04	6.00 ± 0.71	0.00
	0.08	3.20 ± 0.40**	44.83
	0.16	0.60 ± 0.49**	89.66
11	0.02	6.40 ± 0.55	0.00
	0.04	4.00 ± 1.00**	31.03
	0.08	1.40 ± 0.55**	75.86
	0.04	5.80 ± 0.84	0.00
12	0.08	3.80 ± 0.84**	34.48
	0.16	0.60 ± 0.55**	89.66
Oseltamivir	0.01	0.00 ± 0.00**	100.00
	Virus control	5.80 ± 0.84	/
	Norm-control	0.00 ± 0.00	/

**P < 0.01 compared with the virus control; *P < 0.05 compared with the virus control

### CPE reduction in MDCK cells

In the first set of experiments, the cytotoxicity of the compounds was measured using the MTS-based assay. It was found that each compound reduced the viability of MDCK cells with the CC_50_ values (corresponding to a 50% cytotoxic effect after 48 h of the inhibitor treatment) lower than oseltamivir (Table 3). No morphological alternations, loss of cell viability, or modification of cell multiplication rates could be observed in the cells treated with *B. striata* compounds with selected working doses of each compound.

**Table 3:** Antiviral activities of phenanthrenes from *Bletilla striata* in MDCK cells

Compound	CC50	Simultaneous treatment		Post-treatment	
	(μM)^a^	IC50 (μM)^b^	SI^c^	IC50 (μM)^b^	SI^c^
1	200.8 ± 18.6	-	-	-	-
2	67.9 ± 13.1	28.6 ± 4.3	2.3	31.4 ± 2.1	2.2
3	50.3 ± 6.2	20.4 ± 3.2	2.5	22.6 ± 1.8	2.2
4	80.0 ± 20.1	14.6 ± 2.4	5.5	18.4 ± 3.1	4.3
5	96.8 ± 15.6	-	-	-	-
6	106.4 ± 21.2	33.8 ± 2.7	3.1	37.3 ± 3.2	2.8
7	129.5 ± 15.6	28.5 ± 2.8	4.5	31.2 ± 2.2	4.1
8	110.6 ± 17.3	-	-	-	-
9	160.3 ± 20.4	-	-	-	-
10	141.6 ± 25.0	43.3 ± 5.3	3.3	42.3 ± 3.9	3.4
11	118.6 ± 19.6	38.6 ± 2.9	3.1	35.2 ± 3.7	3.4
12	115.7 ± 16.8	-	-	-	-
Oseltamivir	598.8 ± 62.1	4.9 ± 0.9	122.2	0.9 ± 0.2	665.3

^a^CC50: 50% cytotoxic concentration

^b^IC50: 50% inhibitory concentration

^c^SI: selective index; CC50mean/IC50mean

-: The results showed that antiviral effects against A/Sydney/5/97(H3N2) were less than 50% inhibition

**Fig. 3:**
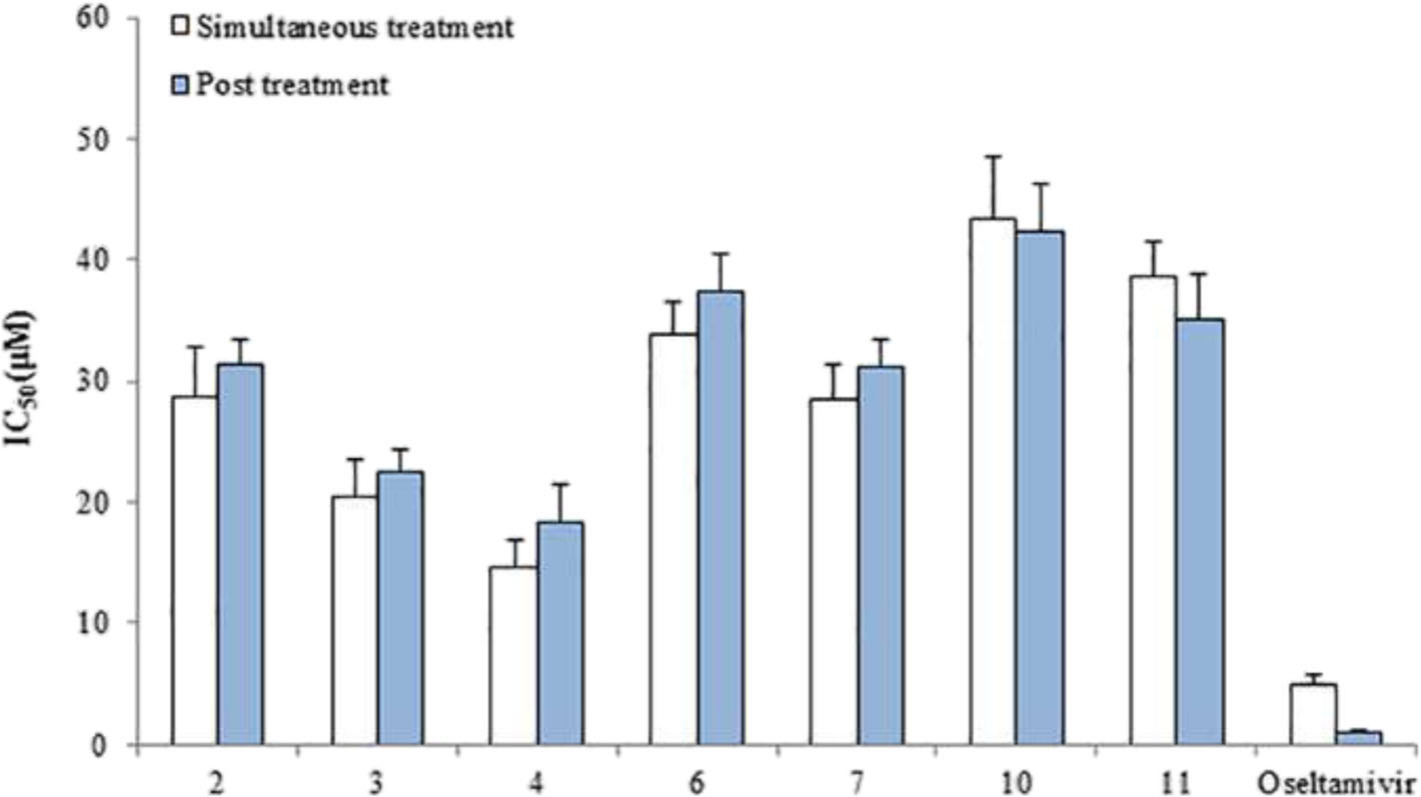
Determination of the IC_50_ of phenanthrenes from *Bletilla striata* by the cytopathic effect (CPE) reduction assay in Madin-Darby canine kidney epithelial cells.. *No significant difference between the two treatments

The MTS-based CPE reduction assay of the MDCK model was used to confirm the antiviral activity. The antiviral effect of the compounds was quantified using the selectivity index (SI), which was the ratio of CC_50mean_ versus IC_50mean_ values (Table 3). The data from the CPE reduction assay demonstrated that *B. striata* compounds exhibited inhibitory antiviral effects in two groups: simultaneous treatment and post-treatment. In the simultaneous treatment assay, compounds 2, 3, 4, 6, 7, 10, and 11 exhibited inhibitory activities against the influenza virus with IC_50_ values ranging from 14.6 ± 2.4 to 43.3 ± 5.3 μM, and compound 4 showed the maximum inhibitory activity (Fig. 3). The IC_50_ values of *B. striata* compounds ranged from 18.4 ± 3.1 to 42.3 ± 3.9 μM in the post-treatment assay (Fig. 3). The compound 4 showed the most potent activity with the SI of 5.5, though it was lower than the value of oseltamivir at 122.2.

### Hemagglutination inhibition of phenanthrenes from *Bletilla striata*

**Fig. 4:**
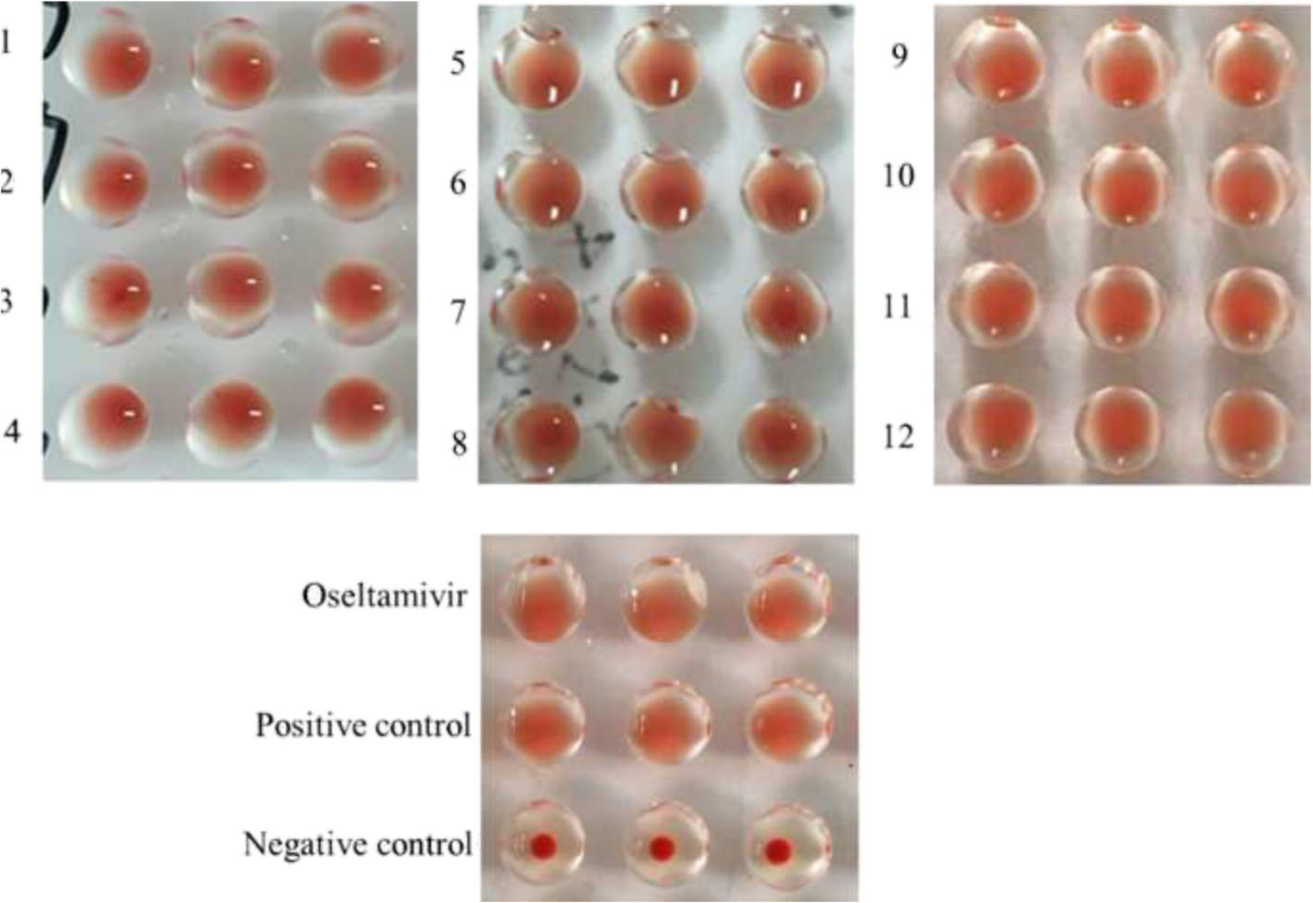
Hemagglutination inhibition of phenanthrenes from *Bletilla striata*. The hemagglutination inhibition assay was used to test the hypothesis that *B. striata* compounds interfere with viral attachment. However, the results showed that the compounds could not inhibit hemagglutination by the influenza virus

The simultaneous treatment assay results indicated that treatment with *B. striata* compounds on virus entry abrogated virus infectivity. Hence, the hemagglutination inhibition assay was used to test the hypothesis that *B. striata* compounds interfere with viral attachment. However, the results showed that the compounds could not inhibit hemagglutination by the influenza virus, neither do oseltamivir (Fig. 4).

### Neuraminidase inhibition of phenanthrenes from *Bletilla striata*

The NA activity assay (NA standard curve and the detailed data are shown in Additional file 1, Fig. 5 and Table 4) was performed to detect any antiviral effect of *B. striata* compounds and to determine the mechanisms. The results (Table 5) showed that compounds 1, 3, 4, 6, 10, and 11 exhibited an inhibitory effect on the NAs in a dose-dependent manner with IC_50_ values ranging from 16.8 ± 1.6 to 87.5 ± 10.1 μM. Compounds 3, 4, and 6 displayed a higher inhibitory activity against the NAs. As a known NA inhibitor, oseltamivir showed a much lower IC_50_ of 0.3 ± 0.02 μM.

**Fig. 5:**
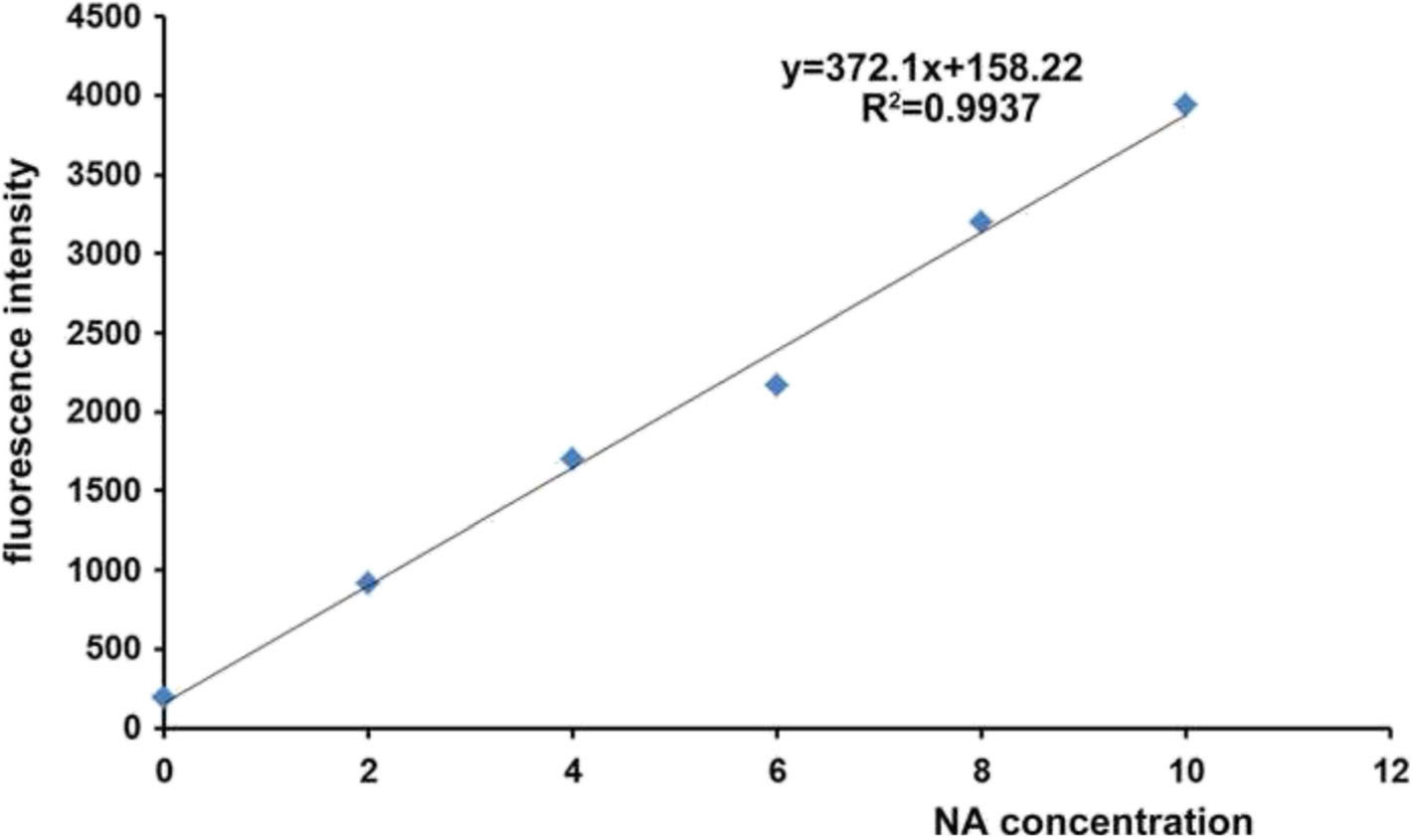
Neuraminidase standard curve performed to detect any antiviral effect of *B. striata* compounds

**Table 4:** NATA for NA standard curve

NA	Fluorescence intensity				SD
(ng/mL)	1	2	3	mean	
0	185.2	192.2	193.7	190.4	4.5
2	982. 0	826.0	942.0	916.7	81.0
4	1734.0	1780.0	1592.0	1702.0	98.0
6	2190.0	2190.0	2121.0	2167.0	39.8
8	3304.0	3201.0	.091.0	3198.7	106.5
10	3961.3	3892.5	3958.9	3937.6	39.0

**Table 5:** NA inhibitory activity of phenanthrenes from *Bletilla striata*

Compound	IC50 (μM)
1	72.6 ± 6.5
2	-
3	16.8 ± 1.6
4	21.7 ± 2.9
5	-
6	16.1 ± 2.6
7	-
8	-
9	-
10	87.5 ± 10.1
11	57.6 ± 5.9
12	-
Oseltamivir	0.3 ± 0.02

-: Inhibitory effect on the NA was less than 50%.

### Inhibition of viral RNA synthesis in MDCK cells

**Fig. 6:**
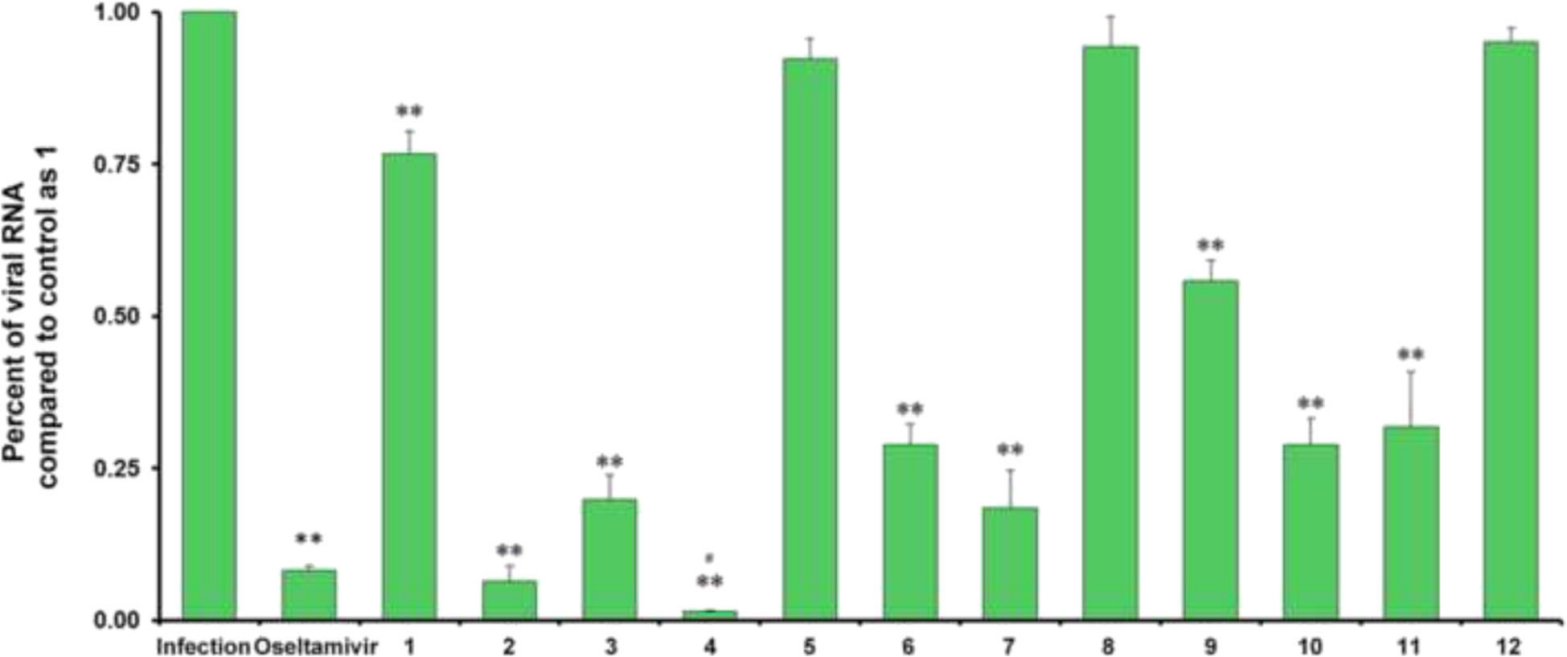
Expression levels of influenza virus RNA normalized to GAPDH. Quantitative RT-PCR showed a reduction in matrix protein mRNA transcription in cell treated with the *B. striata* compounds (8 μM) compared with the untreated infected cells. **P < 0.01 compared with the untreated infected cell group. #*P* < 0.05 compared with the oseltamivir treated group

The synthesis of influenza viral matrix protein mRNA was compared between untreated infected cells and infected cells treated with the compounds to identify any inhibitory effect of the compounds on influenza virus replication. RNA extraction was performed 18 h after influenza virus infection, and the levels of intracellular influenza RNA were measured. Quantitative RT-PCR showed a reduction in matrix protein mRNA transcription in cell treated with the *B. striata* compounds (8 μM) compared with the untreated infected cells (Fig. 6). Compounds 1, 2, 3, 4, 6, 7, 9, 10, and 11 showed a stronger inhibitory effect, with the compound 4 most efficient, even more powerful than oseltamivir. These results indicated that the blockage of virus replication was one of the mechanisms by which *B. striata* compounds exerted antiviral effects.

## Discussion

**Fig. 7:**
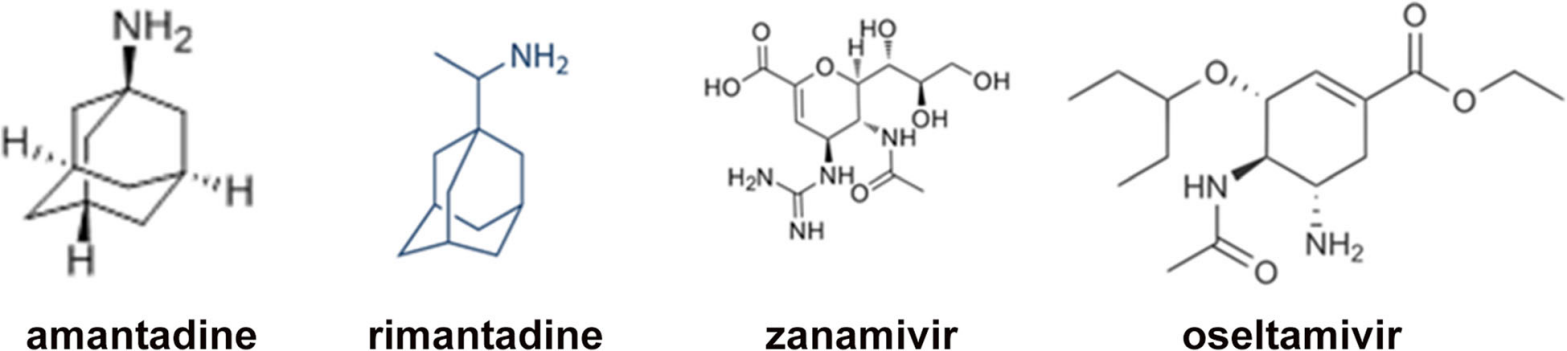
Structures of the main drugs used against influenza (amantadine, rimantadine, zanamivir, and oseltamivir)

Influenza remains a serious problem in many countries, with a long history of outbreaks, epidemics, and recent pandemic [1–4]. With developments in viral biology, more novel antiviral strategies targeting these viruses, such as HA, M2 ion channel protein, RNA-dependent RNA polymerase (RdRp), NP, NS, and NA, have been developed. The two main classes of antiviral drugs clinically used against influenza are inhibitors of the viral M2 protein, such as amantadine (Symmetrel) and rimantadine (Flumadine), or neuraminidase inhibitors, such as zanamivir (Relenza) and oseltamivir (Tamiflu) (Fig.7)

.

**Fig. 8:**
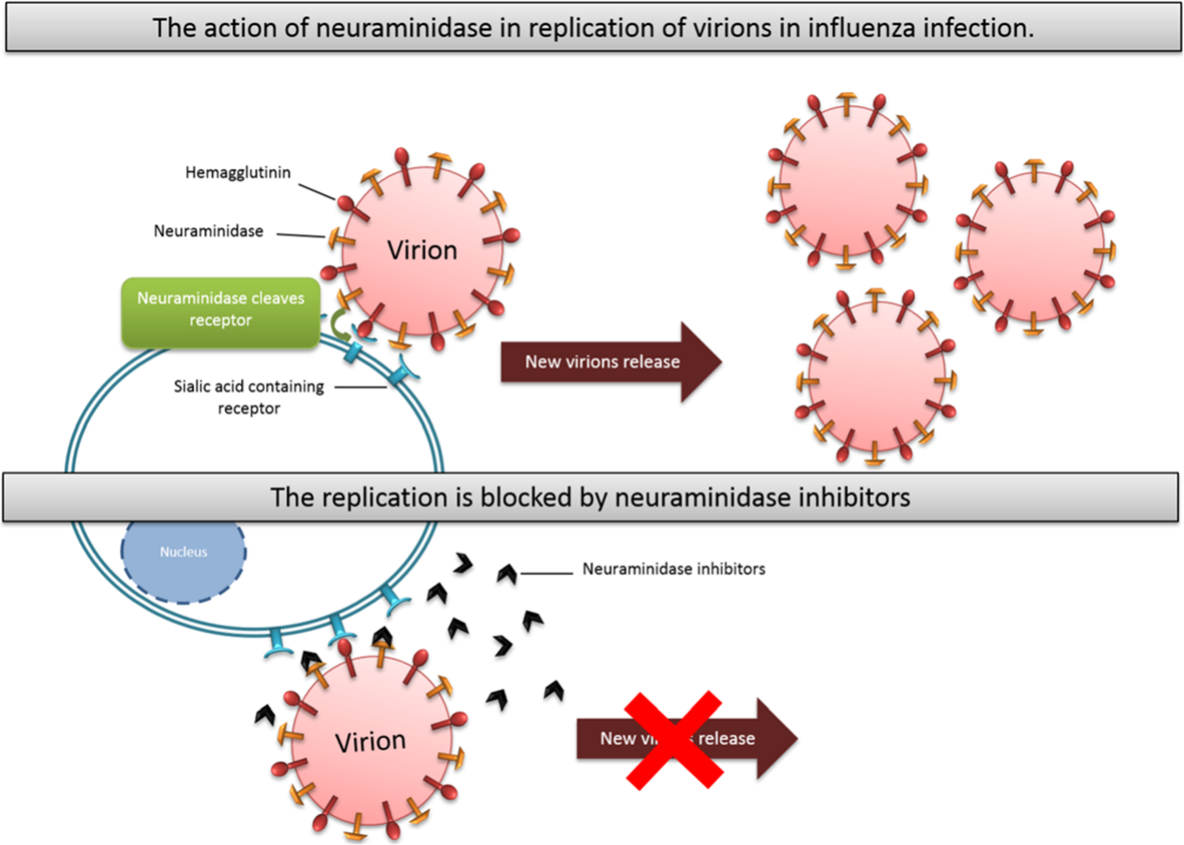
Mechanism of action of neuraminidase inhibitors

M2 protein inhibitor amantadine consists of an adamantane backbone that has an amino group substituted at one of the four methyne positions, and rimantadine is a closely related derivative of adamantane with similar biological properties (Fig. 8). According to the US Centers for Disease Control and Prevention, 100% of seasonal H3N2 and 2009 pandemic flu samples tested have shown resistance to adamantanes, and amantadine is no longer recommended for the treatment of influenza in the United States. A new M2 protein inhibitor is urgently needed.

The M2 channel transports protons from the vacuolar space into the interior of the virion. Acidification of the interior results in the dissociation of ribonucleoproteins and the onset of viral replication. Amantadine and rimantadine function in a mechanistically identical fashion in entering the barrel of the tetrameric M2 channel and blocking the pore function (i.e., proton translocation) [32, 33]. Resistance to the drug class is a consequence of mutations to the pore-lining residues of the channel, leading to the inability of the sterically bulky adamantane ring that both share in entering in their usual way into the channel.

Neuraminidase inhibitor serves as a competitive inhibitor of the activity of the viral neuraminidase (NA) enzyme on SA found in glycoproteins on the surface of normal host cells. By blocking the activity of the enzyme, oseltamivir prevents new viral particles from being released through the cleaving of terminal SA on glycosylated hemagglutinin (Fig. 7, Wikipedia). The discovery of the first designed influenza virus neuraminidase inhibitor and anti-influenza drugs zanamivir and, subsequently, oseltamivir has now inspired a number of continuing efforts toward the discovery of next-generation anti-influenza drugs [34].

Phenanthrene is a polycyclic aromatic hydrocarbon composed of three fused benzene rings. The name “phenanthrene” is a composite of phenyl and anthracene. Most natural phenanthrenes originate biosynthetically from stilbenoids via oxidative coupling of the aromatic rings. They have demonstrated a variety of pharmacological effects, such as antiallergic, anti-inflammatory, antimicrobial, cytotoxic, and spasmolytic activities. Further C-C coupling of two phenanthrenes by 1–1′, 1–3′, 1–8′, or 3–3′ linkages produces rarely occurring biphenanthrenes, which show interesting atropisomeric features due to the hindered rotation of the linkage bond when -OH or -OMe groups are present nearby. Some synthetic chiral biphenanthrenes have been applied as effective enantioselective catalysts and ligands, while the atropisomeric features of many natural biphenanthrenes are not always discussed fully in scientific publications. The optically active naturally occurring biphenanthrene blestriarene C was shown to undergo rapid racemization even in daylight [35, 36].

Some compounds used to fight viral infections are based on the phenanthrene core, including compounds against plant viruses [37, 38] and human viruses [39, 40]. The secretion of phenanthrenes is a natural defense mechanisms of plants against fungal infection [40]. Phenanthrenes from *B. striata* have some efficacy against Gram-positive bacteria, but only weak efficace against fungi [41]. The phenanthrenes of Taxol communis have some efficacy against the vesicular stomatitis virus and the human rhinovirus serotype 1B [39]. A phenanthrene derivative showed some efficacy against coronaviruses [42]. The antiviral property of phenanthrenes from *B. striata* against the influenza A virus was investigated in this study using embryonated hen eggs, and it was confirmed that compounds 1, 2, 3, 4, 6, 9, 10, 11, and 12 exerted significant inhibitory effects. In MDCK models, compounds 2, 3, 4, 6, 7, 10, and 11 exhibited CPE reduction activities. The study showed that phenanthrenes from *B. striata* had strong anti-influenza viral activity. Furthermore, the HA, matrix protein, and NA were employed as targets for the study of possible mechanisms.

*B. striata* compounds 2, 3, 4 6, 7, 10, and 11 served as matrix protein inhibitors and resulted in the reduction of mRNA transcription. The function-structure relationship of these compounds and whether they interfere with the M2 proton channel need further exploration.

Compounds 1, 3, 4, 6, 10, and 11 exhibited an inhibitory effect on NA; compounds 3, 4, and 6 displayed a higher inhibitory activity. NA is an exoglycosidase that destroys the HA receptor by cleaving the α(2,6)- or α(2,3)-ketosidic linkage that exists between a terminal SA and a sugar residue of the N-acetylneuraminic acid (Neu5Ac)-containing receptor on the surface of the host cells [5]. Neuraminidase inhibitors commonly interfere with the enzyme activity by mimicking the natural substrate and fitting into the active site of the neuraminidase enzyme. The binding efficiency of the compound with the neuraminidase is the major determinant of the inhibitory activity. The structure-activity relationship of new neuraminidase inhibitors should be proved for better molecular properties and higher efficiency against neuraminidase receptor compared with zanamivir or oseltamivir.

In the present study, compounds 4, 6, 9, and 12 showed the same efficacy in the egg model. The reasons for the similar efficacy are presently unknown. Indeed, these four compounds do not share the same molecular structure. The results could have been biased by the small number of eggs used in the experiments. There is also a possibility that these compounds share a mechanism that has not been studied here. In addition, some compounds inhibited the influenza virus through two mechanisms, but the reasons for this dual inhibition are currently unknown. Additional studies are necessary to better characterize these compounds.

## Conclusions

This study indicated that diphenanthrenes (compounds 2–8 and 11) had stronger inhibitory activity compared with monophenanthrenes (compounds 1, 9, 10, and 12). In MDCK models, the antiviral effects of most monophenanthrenes were less than 50% except compound 10, while the effects of diphenanthrenes were all higher than 50% and IC_50_ could be calculated. This might be because of the hydroxyl group, which impacts the hydrophilicity/hydrophobicity of the compound. All diphenanthrenes share more hydroxyl groups. However, how the hydroxyl group affects the antiviral activity still needs further investigation. Compounds 2, 3, 4 6, 7, 10, and 11 served as matrix protein inhibitors and resulted in the reduction of mRNA transcription. Compounds 1, 3, 4, 6, 10, and 11 exhibited an inhibitory effect on NA. Additional studies are required to elucidate the exact mechanisms of these compounds.

## Additional file

Additional file 1: The additional file are data of virus HA titer of allantoic fluid from embryonated hen eggs to assay antiviral activity and data for NA standard curve. (DOCX 2775 kb)

## Abbreviations

CPE: Cytopathic effect
DPPH: Diphenyl-1-picrylhydrazyl
FBS: Fetal bovine serum
HA: Hemagglutinin
HAU: Hemagglutination units
HPLC: High-performance liquid chromatography
MDCK: Madin-Darby canine kidney
MEM: Minimum essential medium
NA: Neuraminidase
NPs: Nucleoproteins
PBS: Phosphate-buffered saline
RBC: Red blood cells
RdRp: RNA-dependent RNA polymerase
