# Supplementary material for: Antiviral activity of phenanthrenes from the medicinal plant Bletilla striata against influenza A virus
Source: BMC Complement Altern Med. 2017 May 22;17:273. doi: 10.1186/s12906-017-1780-6 (PMC5441103; doi:10.1186/s12906-017-1780-6)
Supplement: Additional file 1: — The additional file are data of virus HA titer of allantoic fluid from embryonated hen eggs to assay antiviral activity and data for NA standard curve. (DOCX 2775 kb) [file 12906_2017_1780_MOESM1_ESM.docx]

**The spectra for all the 12 phenanthrenes from the medicinal plant *Bletilla striata***

Compound 1

**
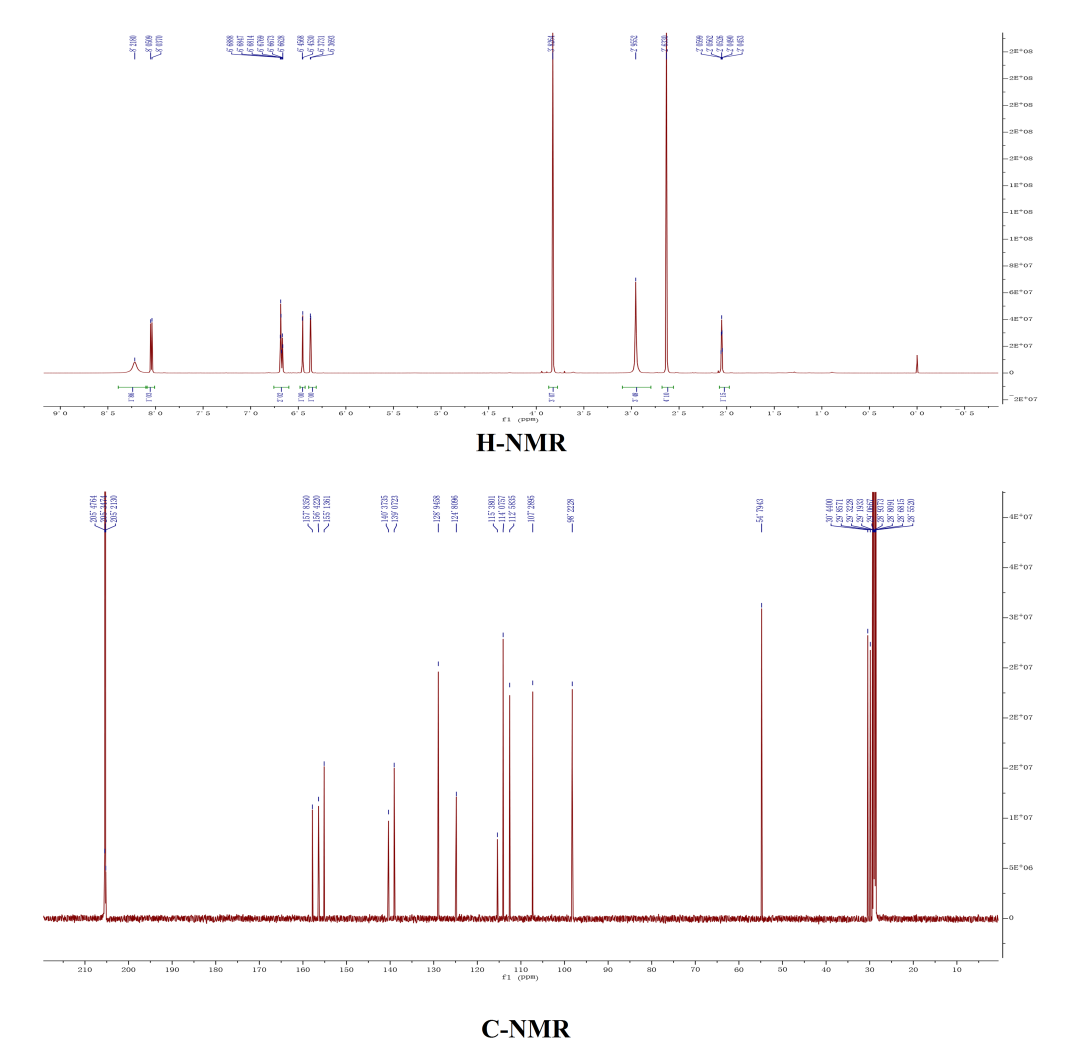
**

Compound 2

**
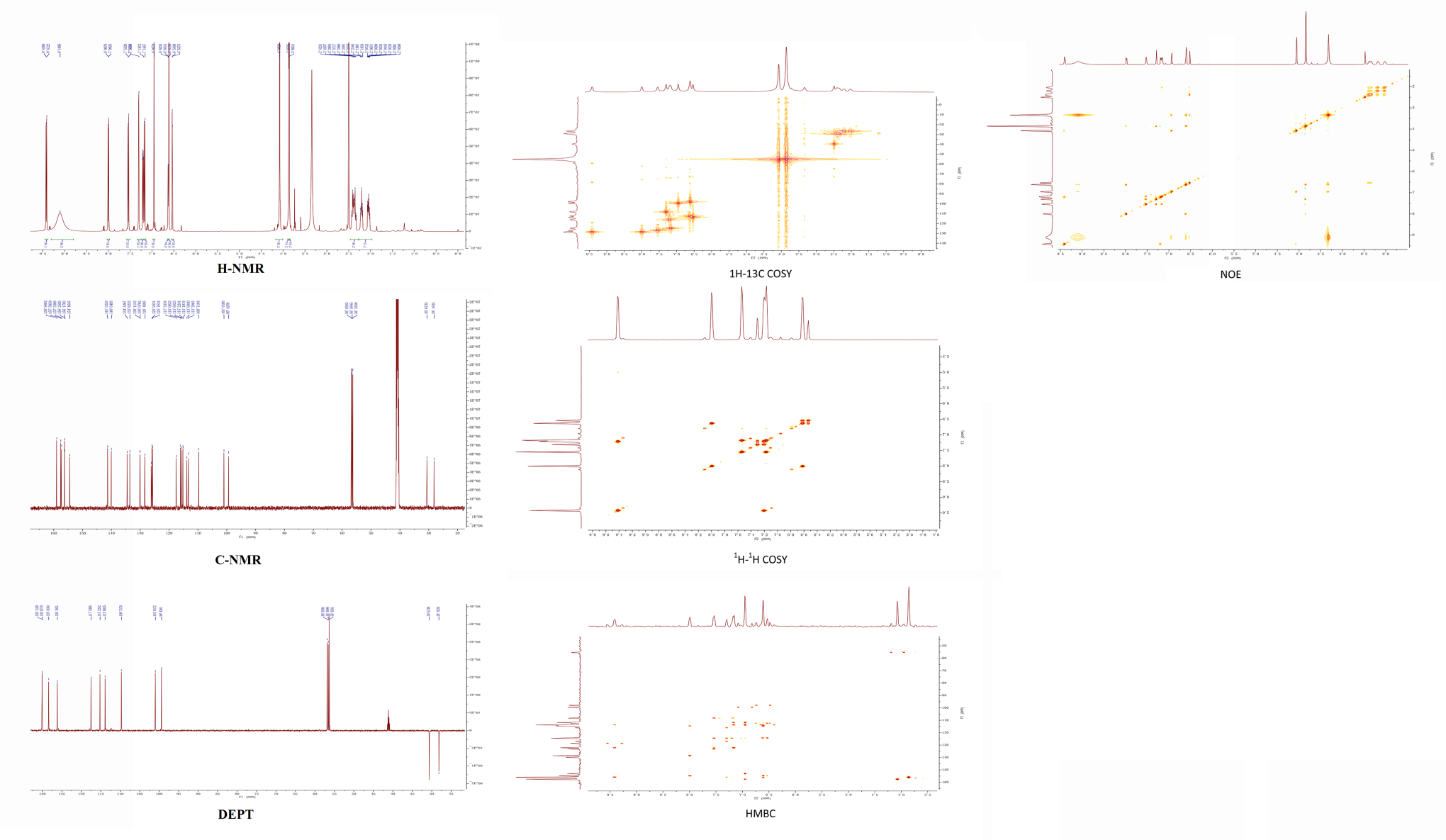
**

Compound 3


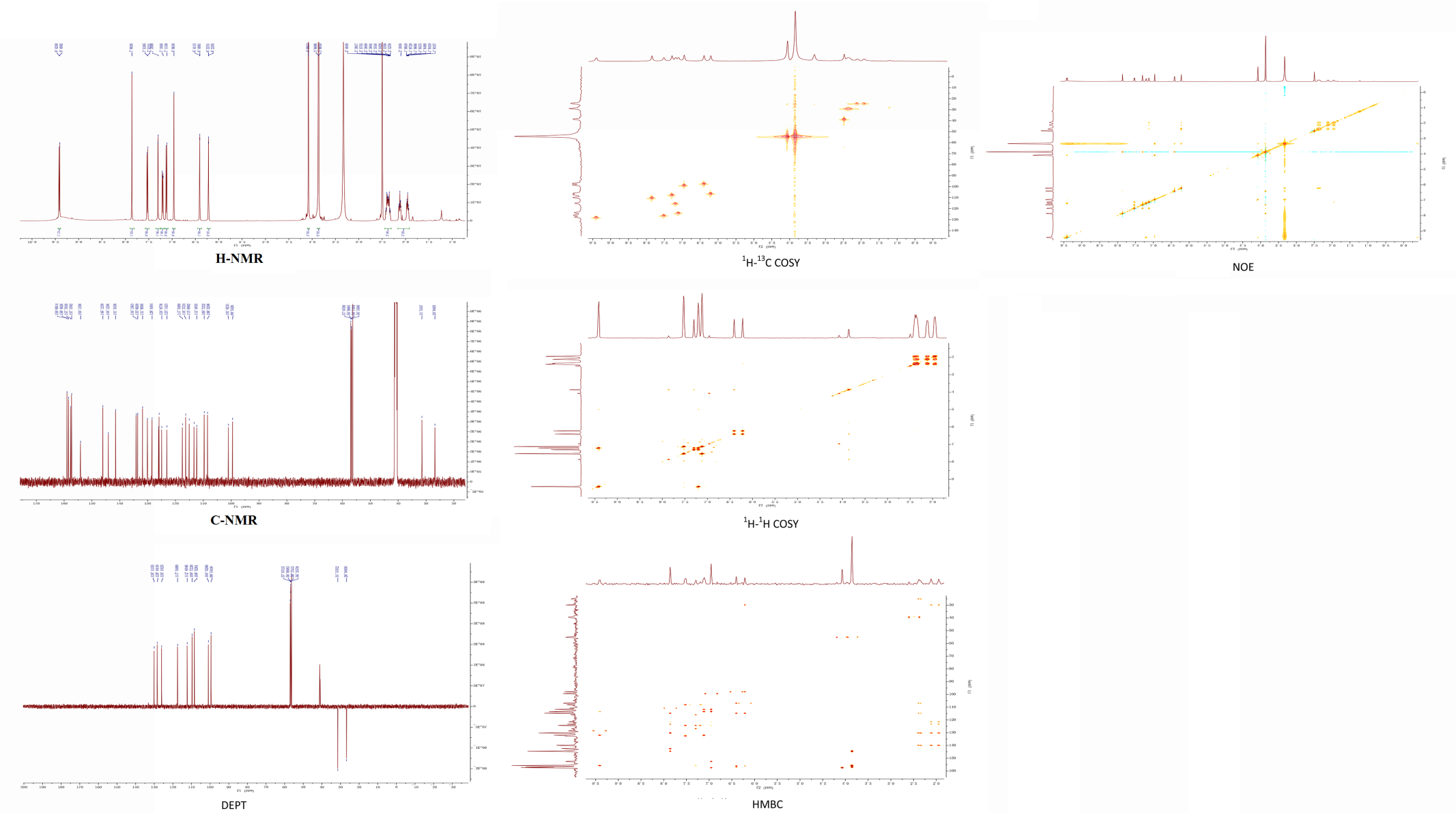


Compound 4


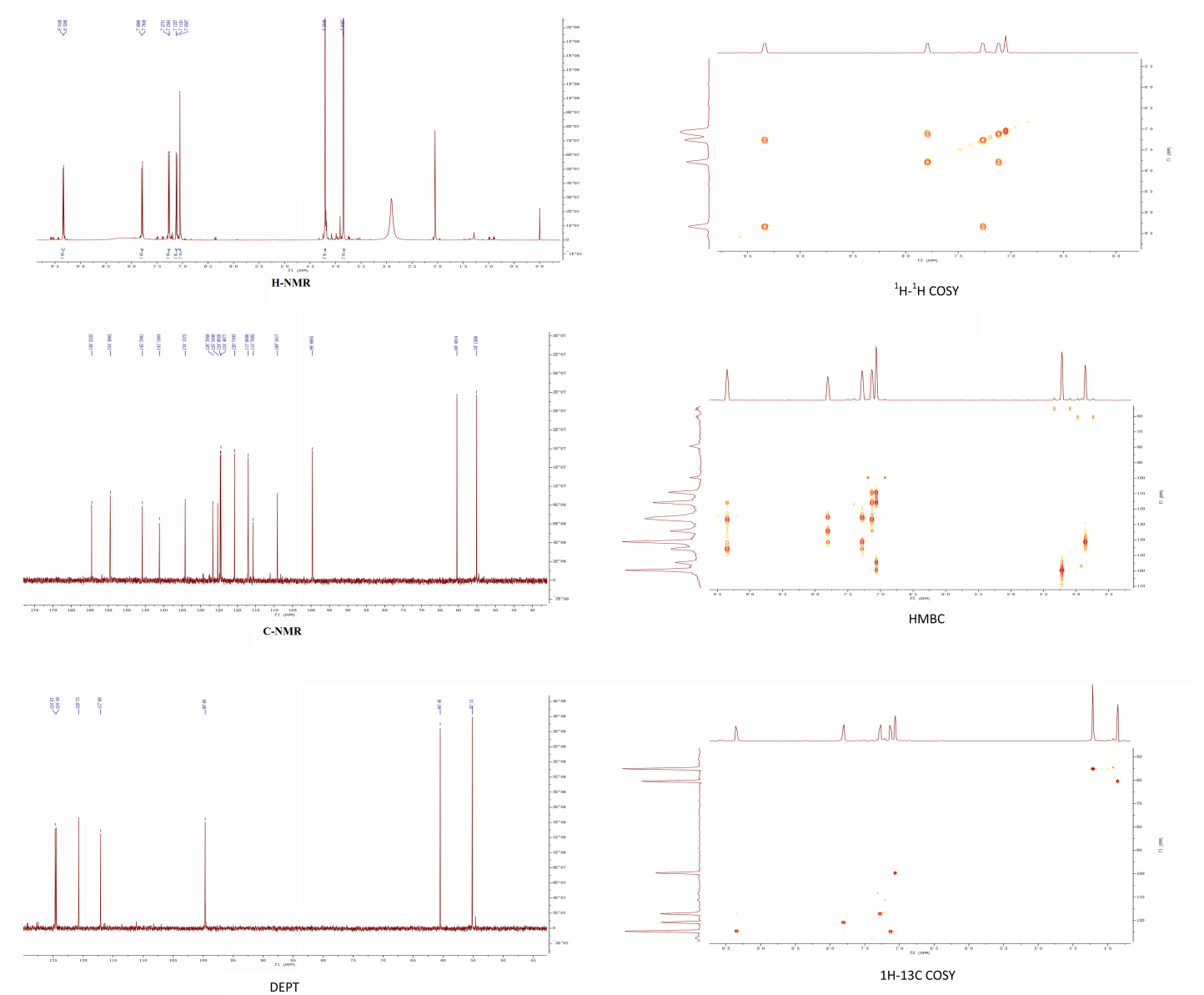


Compound 5


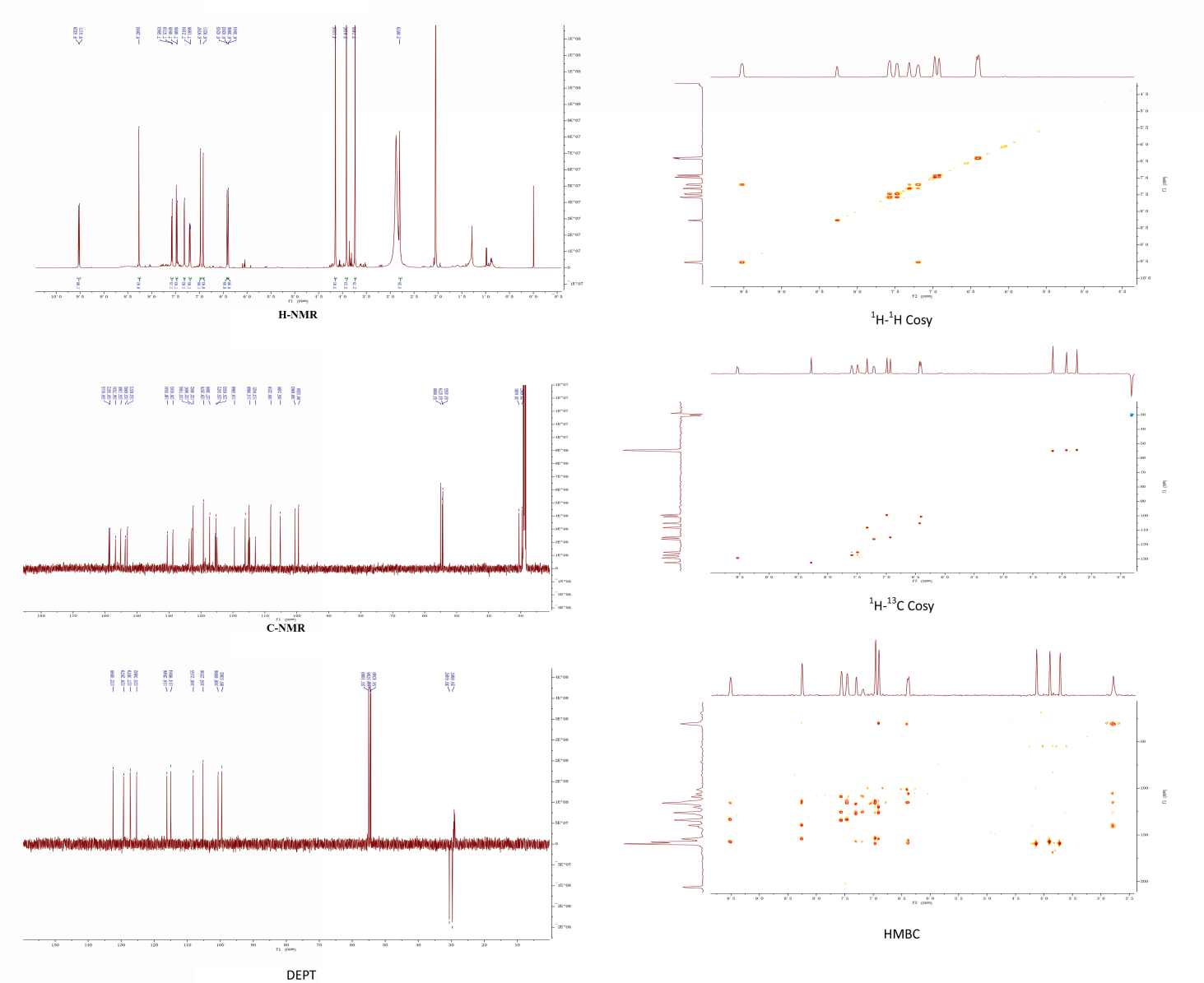


Compound 6


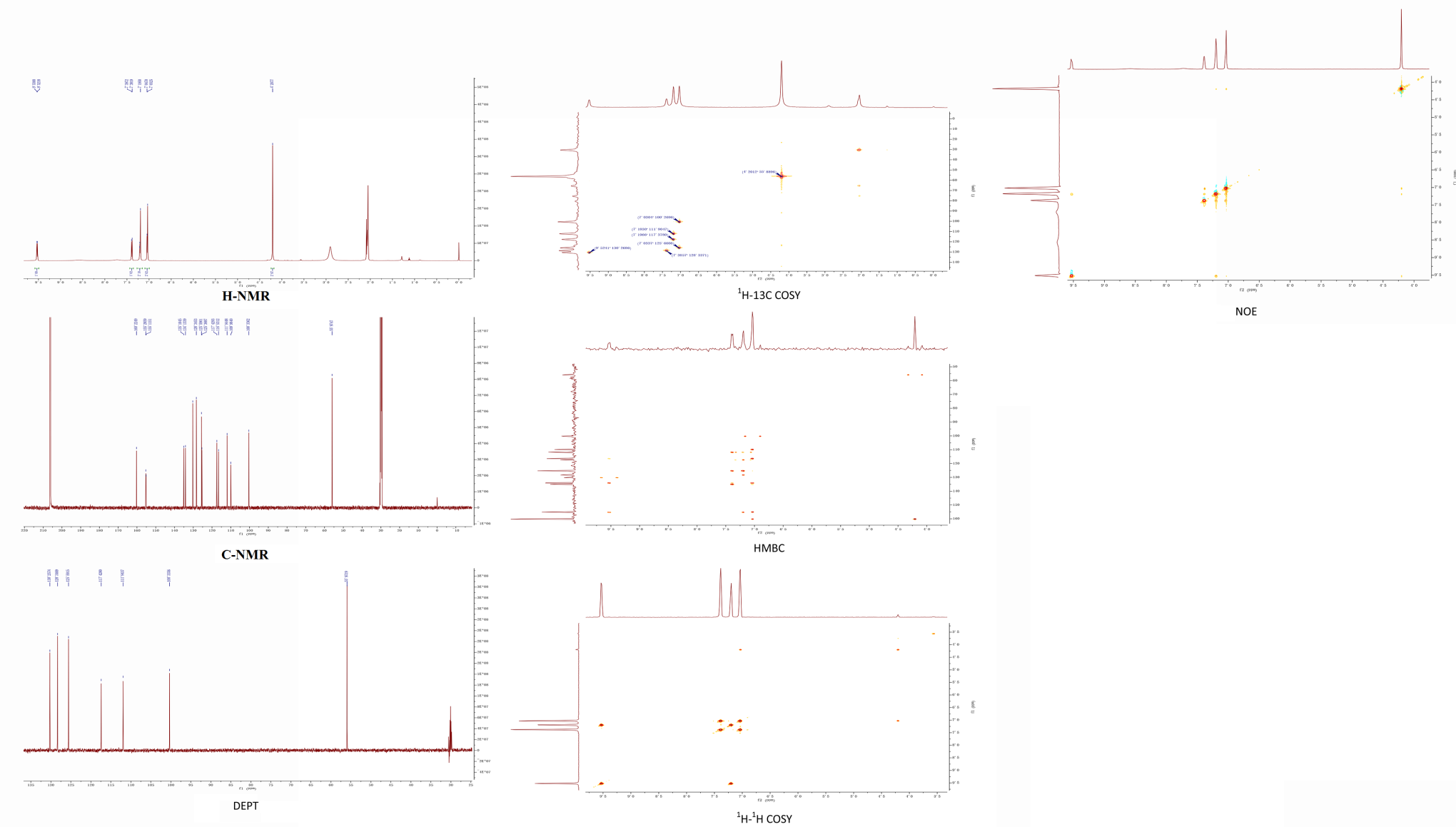


Compound 7


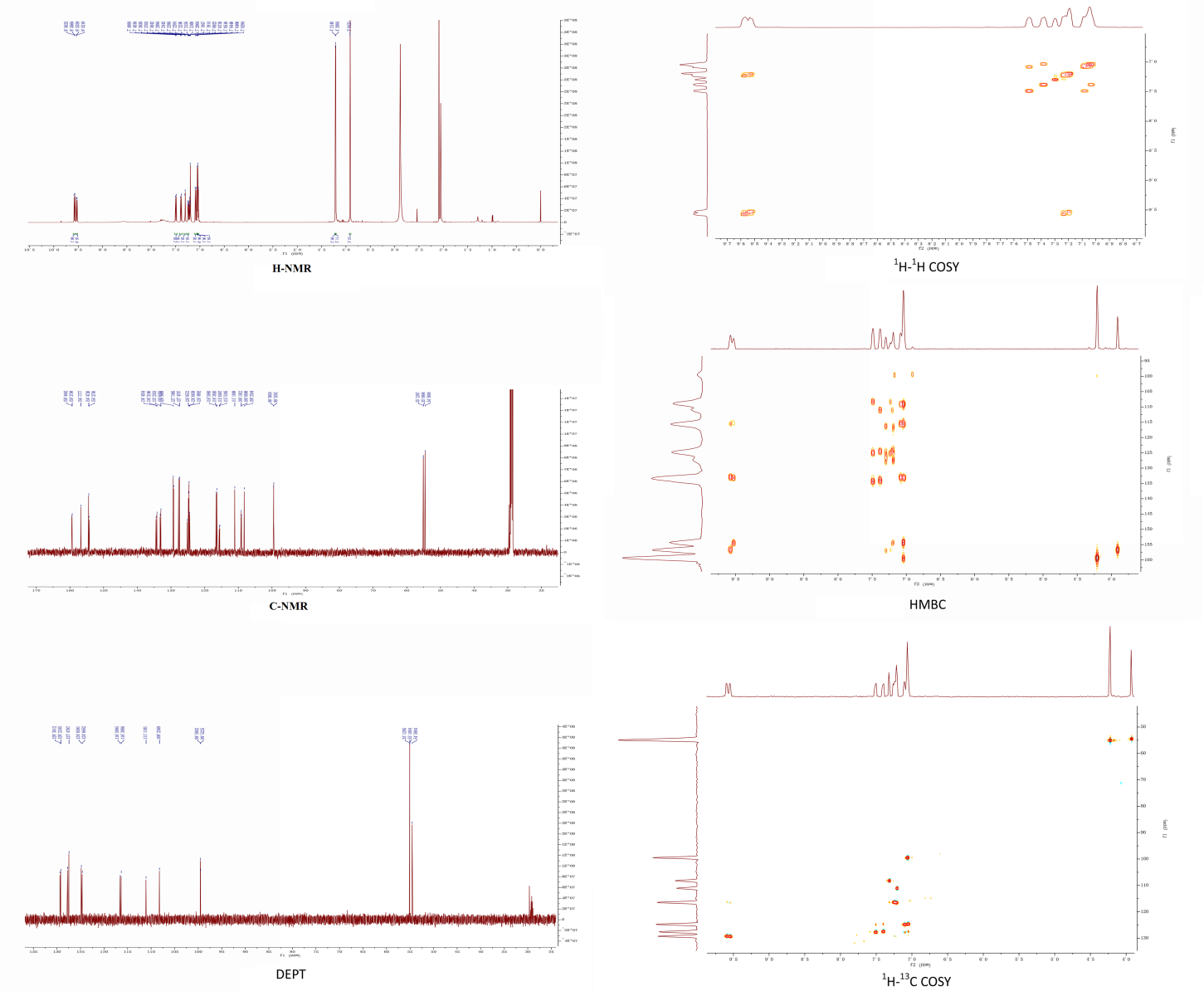


Compound 8


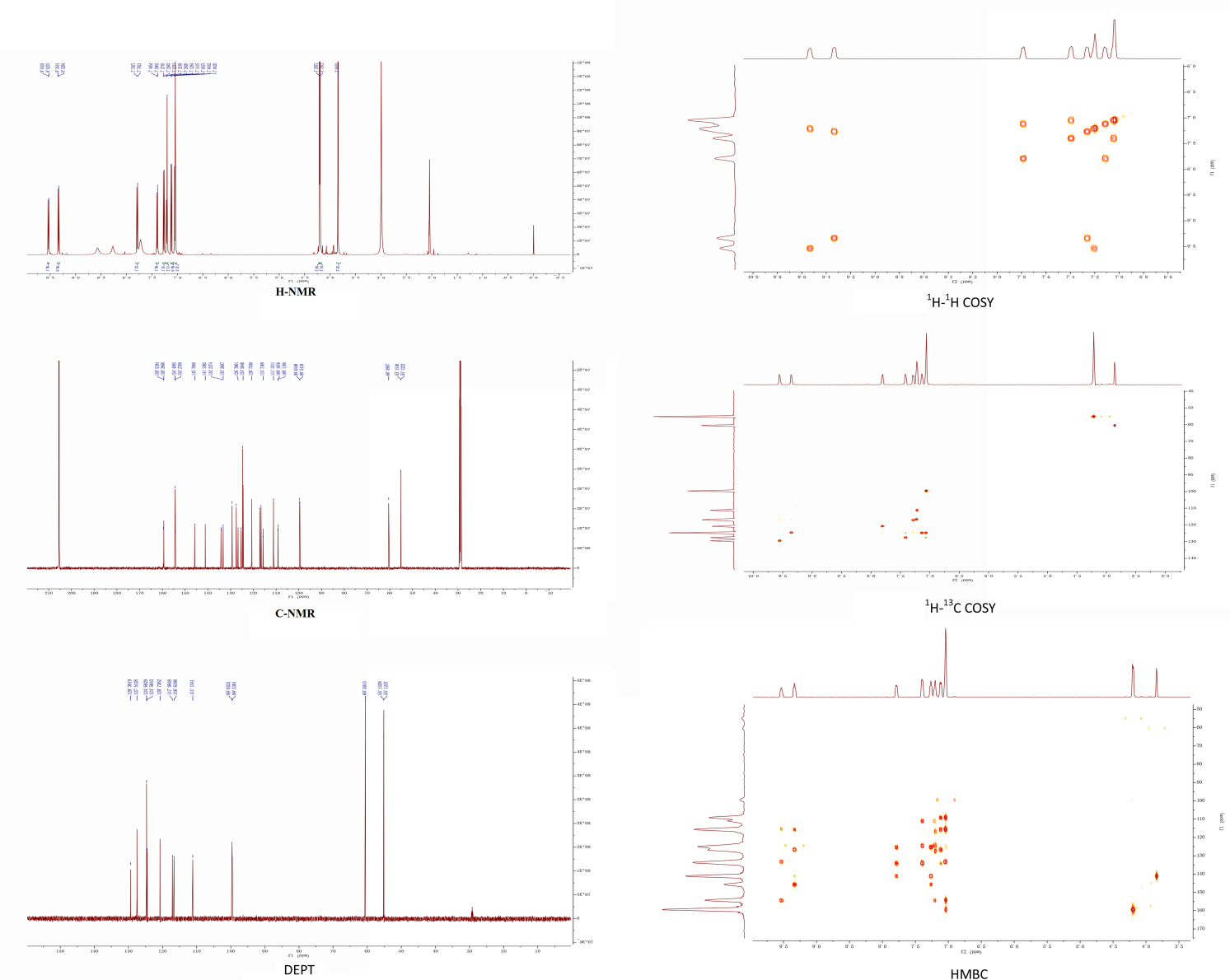


Compound 9


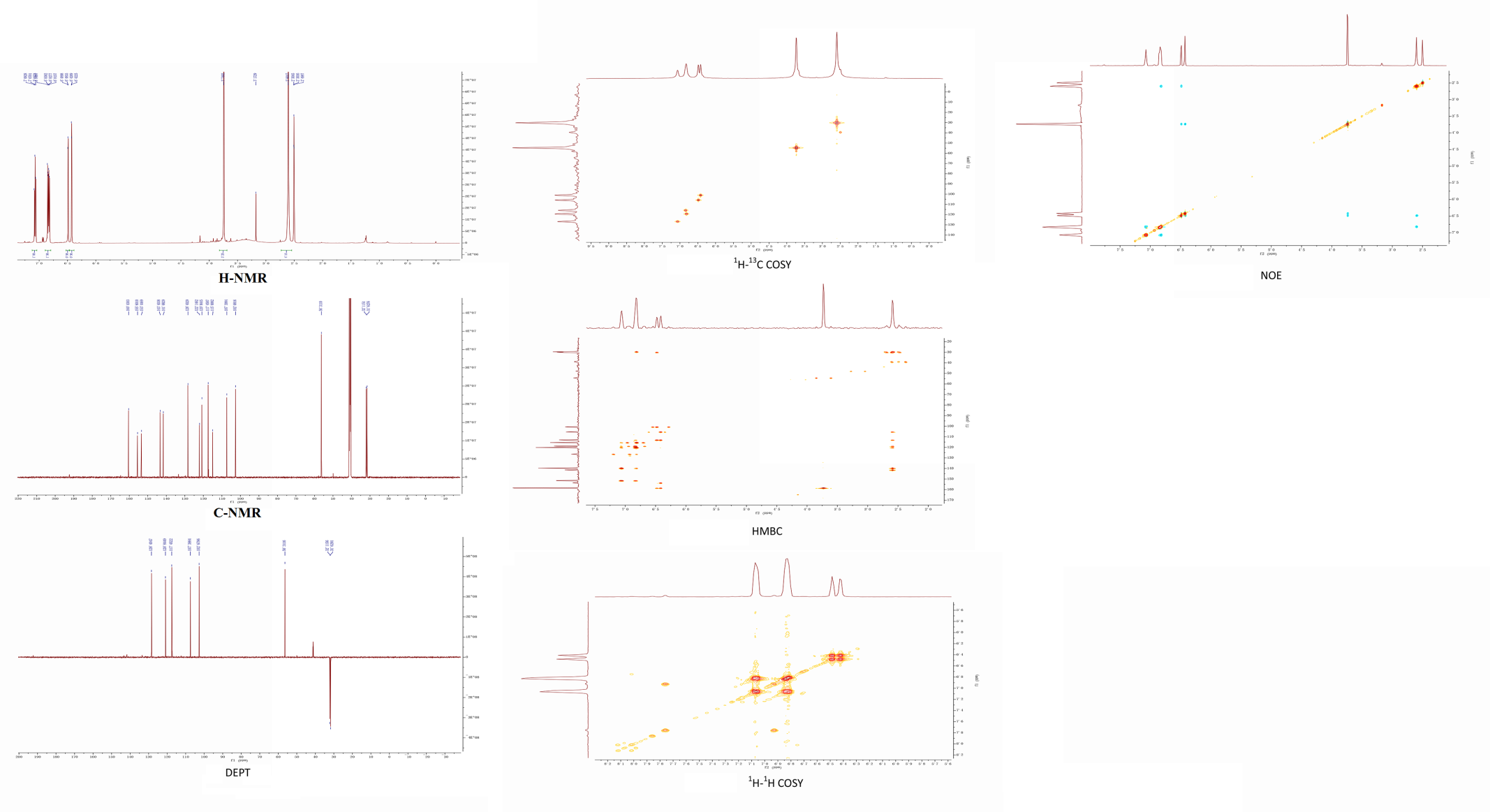


Compound 10


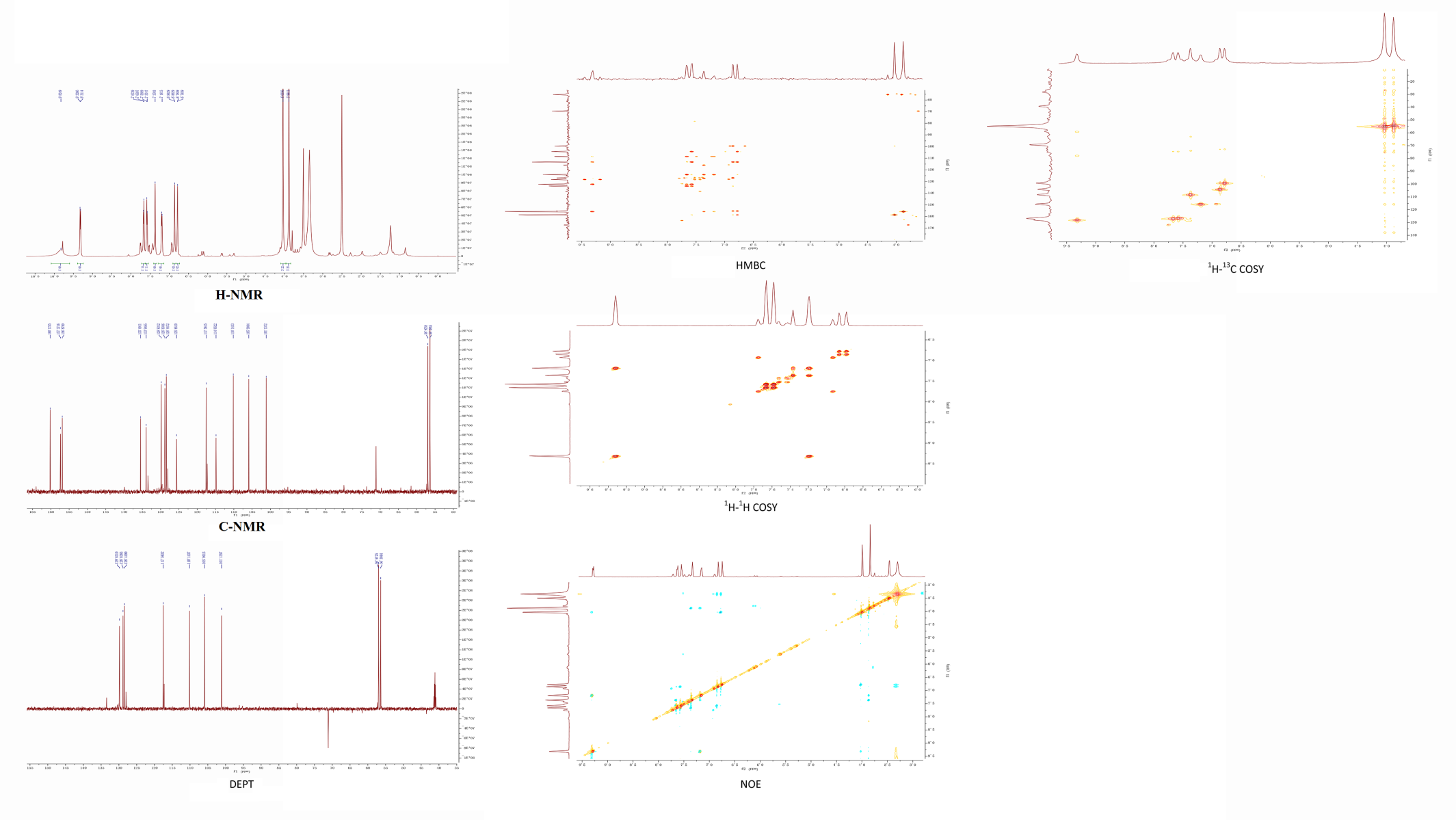


Compound 11


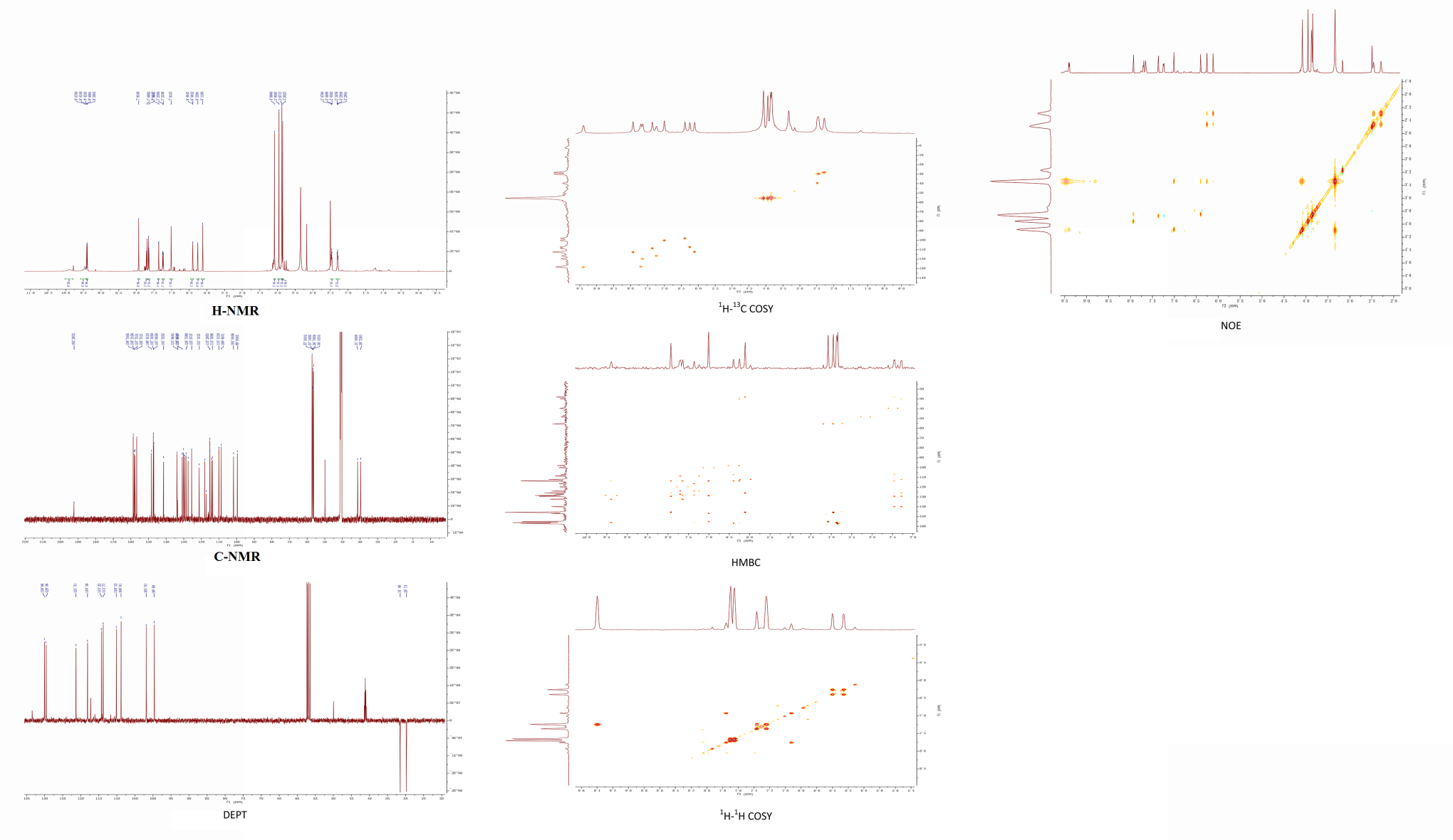


Compound 12


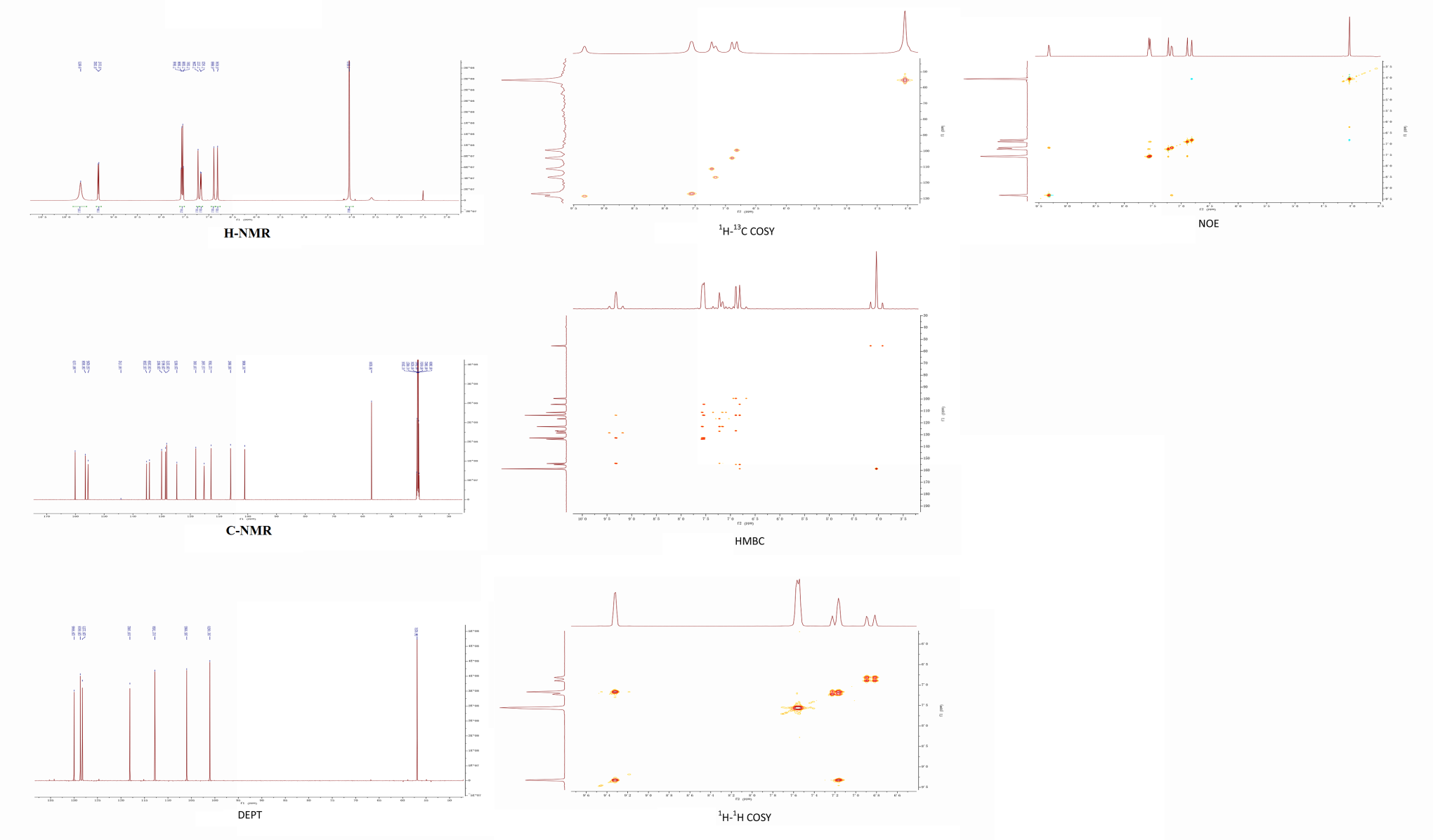


**The spectral data for all the 12 phenanthrenes from the medicinal plant *Bletilla striata***

Compound 1

|  | Position | δ (ppm) | J (Hz) |
| --- | --- | --- | --- |
| ^1^H-NMR | H-9,10 | 2.63(4H,s) |  |
|  | 4-OCH3 | 3.83(3H,s) |  |
|  | H-1 | 6.37(1H,d) | 2.3 |
|  | H-3 | 6.45(1H,d) | 2.3 |
|  | H-6 | 6.68(1H,d) | 2.3 |
|  | H-8 | 6.67(1H,dd) | 8.4,2.6 |
|  | H-5 | 8.04(H,d) | 8.4 |
| ^13^C-NMR | C-2 | 157.84 |  |
|  | C-7 | 156.42 |  |
|  | C-4 | 155.14 |  |
|  | C-10a | 140.37 |  |
|  | C-8a | 139.07 |  |
|  | C-5 | 128.95 |  |
|  | C-4a | 124.81 |  |
|  | C-5a | 115.38 |  |
|  | C-6 | 114.08 |  |
|  | C-8 | 112.58 |  |
|  | C-1 | 107.29 |  |
|  | C-3 | 98.22 |  |
|  | -OCH3 | 54.79 |  |
|  | C-9 | 30.44 |  |
|  | C-10 | 29.86 |  |

Compound 2

|  | Position | δ (ppm) | J (Hz) |
| --- | --- | --- | --- |
| ^1^H-NMR | H-9’,10’ | 2.15-2.35 (4H,m) |  |
|  | 4’-OCH_3_ | 3.86(3H,s) |  |
|  | 7-OCH_3_ | 3.88(3H,s) |  |
|  | 4-OCH_3_ | 4.08(3H,s) |  |
|  | H-8’ | 6.54(1H,d) | 2.1 |
|  | H-3’ | 6.62(1H,s) |  |
|  | H-6’ | 6.63(1H,dd) | 8.6,2.4 |
|  | H-3 | 6.96(1H,s) |  |
|  | H-10 | 7.17(1H,d) | 9.2 |
|  | H-6 | 7.21(1H,dd) | 9.5,2.9 |
|  | H-8 | 7.31(1H,d) | 2.8 |
|  | H-9 | 7.54(1H,d) | 9.2 |
|  | H-5’ | 8.00(1H,d) | 8.7 |
|  | 2,2’,7’-OH | 9.11(3H,s) |  |
|  | H-5 | 9.42(1H,d) | 9.5 |
| ^13^C-NMR | C-4 | 158.98 |  |
|  | C-7 | 157.99 |  |
|  | C-4’ | 157.34 |  |
|  | C-2 | 156.25 |  |
|  | C-2’ | 156.18 |  |
|  | C-7’ | 154.46 |  |
|  | C-10a’ | 141.33 |  |
|  | C-8a’ | 140.05 |  |
|  | C-10a | 134.54 |  |
|  | C-8a | 133.62 |  |
|  | C-5 | 130.11 |  |
|  | C-5’ | 130.08 |  |
|  | C-9 | 128.41 |  |
|  | C-10 | 126.18 |  |
|  | C-5a’ | 125.95 |  |
|  | C-5a | 125.76 |  |
|  | C-6 | 117.54 |  |
|  | C-8’ | 116.07 |  |
|  | C-4a’ | 115.66 |  |
|  | C-6 | 115.22 |  |
|  | C-4a | 115.19 |  |
|  | C-1’ | 113.90 |  |
|  | C-1 | 113.30 |  |
|  | C-8 | 109.72 |  |
|  | C-3 | 101.02 |  |
|  | C-3’ | 99.43 |  |
|  | 4-OCH_3_ | 56.86 |  |
|  | 7-OCH_3_ | 56.59 |  |
|  | 4’-OCH_3_ | 56.36 |  |
|  | C-9’ | 30.67 |  |
|  | C-10’ | 28.81 |  |

Compound 3

|  | Position | δ (ppm) | J (Hz) |
| --- | --- | --- | --- |
| ^1^H-NMR | H-9’,10’ | 1.92-2.45(4H,m) |  |
|  | 3’,5’,7-OCH_3_ | 3.87(9H,s) |  |
|  | 4-OCH_3_ | 4.08(3H,s) |  |
|  | H-8’ | 6.22(1H,d) | 1.5 |
|  | H-3’ | 6.41(1H,d) | 1.9 |
|  | H-3 | 6.96(1H,s) |  |
|  | H-10 | 7.12(1H,d) | 9.2 |
|  | H-6 | 7.20(1H,dd) | 9.5,2.8 |
|  | H-8 | 7.30(1H,d) | 2.8 |
|  | H-9 | 7.53(1H,d) | 9.2 |
|  | H-4’ | 7.86(1H,s) |  |
|  | H-5 | 9.42(1H,d) | 9.5 |
| ^13^C-NMR | C-4 | 159.00 |  |
|  | C-5’ | 158.46 |  |
|  | C-7’ | 157.70 |  |
|  | C-7 | 157.34 |  |
|  | C-2 | 154.14 |  |
|  | C-2’ | 146.12 |  |
|  | C-3’ | 144.13 |  |
|  | C-10a’ | 141.54 |  |
|  | C-8a’ | 134.16 |  |
|  | C-10a | 133.60 |  |
|  | C-8a | 131.86 |  |
|  | C-5 | 130.12 |  |
|  | C-9 | 128.44 |  |
|  | C-10 | 126.10 |  |
|  | C-5a’ | 125.92 |  |
|  | C-5a | 124.98 |  |
|  | C-1’ | 123.14 |  |
|  | C-6 | 117.55 |  |
|  | C-4a’ | 116.37 |  |
|  | C-4a | 115.09 |  |
|  | C-1 | 113.36 |  |
|  | C-4’ | 112.41 |  |
|  | C-8 | 109.72 |  |
|  | C-8’ | 108.53 |  |
|  | C-3 | 101.02 |  |
|  | C-6’ | 99.54 |  |
|  | 4-OCH_3_ | 57.07 |  |
|  | 2’,5’,7-OCH_3_ | 56.87,56.75,56.36 |  |
|  | C-9’ | 31.53 |  |
|  | C-10’ | 26.84 |  |

Compound 4

|  | Position | δ (ppm) | J (Hz) |
| --- | --- | --- | --- |
| ^1^H-NMR | 8,8’-OCH_3_ | 3.85 (6H,s) |  |
|  | 2,2’-OCH_3_ | 4.20(6H,s) |  |
|  | H-3,3’ | 7.05(2H,s) |  |
|  | H-10,10’ | 7.12(2H,d) | 9.4 |
|  | H-6,6’ | 7.27(2H,d) | 9.4 |
|  | H-9,9’ | 7.79(2H,d) | 9.4 |
|  | H-5,5’ | 9.34(2H,d) | 9.5 |
| ^13^C-NMR | C-2,2’ | 159.53 |  |
|  | C-4,4’ | 154.49 |  |
|  | C-7,7’ | 145.79 |  |
|  | C-8,8’ | 141.15 |  |
|  | C-8a,8a’ | 134.16 |  |
|  | C-5a,5a’ | 126.71 |  |
|  | C-10a,10a’ | 125.35 |  |
|  | C-10,10’ | 124.66 |  |
|  | C-5,5’ | 124.49 |  |
|  | C-9,9’ | 120.75 |  |
|  | C-6,6’ | 117.09 |  |
|  | C-4a,4a’ | 115.79 |  |
|  | C-1,1’ | 109.16 |  |
|  | C-3,3’ | 99.67 |  |
|  | C-8,8’- OCH_3_ | 60.48 |  |
|  | C-2,2’- OCH_3_ | 55.15 |  |

Compound 5

|  | Position | δ (ppm) | J (Hz) |
| --- | --- | --- | --- |
| ^1^H-NMR | H-9’,10’ | 2.81(4H,s) |  |
|  | 2’-OCH_3_ | 3.74(3H,s) |  |
|  | 7-OCH_3_ | 3.92 (3H,s) |  |
|  | H-1’ | 6.40(1H,d) | 2.5 |
|  | H-3’ | 6.42(1H,d) | 2.5 |
|  | H-8’ | 6.92(1H,s) |  |
|  | H-3 | 6.98(1H,s) |  |
|  | H-6 | 7.20(1H,dd) | 9.5,2.9 |
|  | H-8 | 7.32(1H,d) | 2.9 |
|  | H-10 | 7.48(1H,d) | 9.1 |
|  | H-9 | 7.58(1H,d) | 9.2 |
|  | H-5’ | 8.27(1H,s) |  |
|  | H-5 | 9.53(1H,d) | 9.5 |
| ^13^C-NMR | C-2 | 158.81 |  |
|  | C-2’ | 158.56 |  |
|  | C-7 | 156.75 |  |
|  | C-4’ | 155.14 |  |
|  | C-7’ | 153.69 |  |
|  | C-4 | 153.04 |  |
|  | C-10a’ | 140.55 |  |
|  | C-8a’ | 138.82 |  |
|  | C-10a | 133.75 |  |
|  | C-8a | 132.97 |  |
|  | C-5’ | 132.49 |  |
|  | C-5 | 129.29 |  |
|  | C-9 | 127.31 |  |
|  | C-5a’ | 125.55 |  |
|  | C-10 | 125.35 |  |
|  | C-5a | 125.04 |  |
|  | C-4a’ | 119.60 |  |
|  | C-6 | 116.20 |  |
|  | C-4a | 115.23 |  |
|  | C-8’ | 114.99 |  |
|  | C-1 | 114.81 |  |
|  | C-6’ | 113.04 |  |
|  | C-8 | 108.22 |  |
|  | C-1’ | 105.24 |  |
|  | C-3’ | 100.70 |  |
|  | C-3 | 99.54 |  |
|  | 2-OCH_3_ | 55.10 |  |
|  | 2’-OCH_3_) | 54.58 |  |
|  | 7-OCH_3_ | 54.44 |  |
|  | C-9’ | 30.65 |  |
|  | C-10’ | 29.70 |  |

Compound 6

|  | Position | δ (ppm) | J (Hz) |
| --- | --- | --- | --- |
| ^1^H-NMR | 2,2’-OCH_3_ | 4.21 (6H,s) |  |
|  | H-3,3’ | 7.03(2H,s) |  |
|  | H-10,10’ | 7.05(2H,d) | 9.4 |
|  | H-8,8’ | 7.20(2H,d) | 2.6 |
|  | H-6,6’ | 7.22(2H,dd) | 9.0,2.6 |
|  | H-9,9’ | 7.39(2H,d) | 9.0 |
|  | H-5,5’ | 9.53(2H,d) | 9.0 |
| ^13^C-NMR | C-2,2’ | 160.22 |  |
|  | C-7,7’ | 155.29 |  |
|  | C-4,4’ | 155.12 |  |
|  | C-8a,8a’ | 135.10 |  |
|  | C-10a,10a’ | 134.13 |  |
|  | C-9,9’ | 128.35 |  |
|  | C-10,10’ | 125.59 |  |
|  | C-5a,5a’ | 125.37 |  |
|  | C-8,8’ | 117.43 |  |
|  | C-4a,4a’ | 116.53 |  |
|  | C-6,6’ | 111.94 |  |
|  | C-1,1’ | 109.98 |  |
|  | C-3,3’ | 100.34 |  |
|  | C-2,2’- OCH_3_ | 55.97 |  |

Compound 7

|  | Position | δ (ppm) | J (Hz) |
| --- | --- | --- | --- |
| ^1^H-NMR | 7’-OCH_3_ | 3.91 (3H,s) |  |
|  | 2,2’-OCH_3_ | 4.21 (6H,s) |  |
|  | H-10 | 7.03(1H,d) | 9.1 |
|  | H-3 | 7.04(1H,s) |  |
|  | H-3’ | 7.05(1H,s) |  |
|  | H-10’ | 7.08(1H,d) | 9.1 |
|  | H-8 | 7.20(1H,d) | 2.9 |
|  | H-6 | 7.21(1H,dd) | 9.5,2.9 |
|  | H-6’ | 7.23(1H,dd) | 9.5,2.9 |
|  | H-8’ | 7.30(1H,d) | 2.9 |
|  | H-9 | 7.39(1H,d) | 9.2 |
|  | H-9’ | 7.49(1H,d) | 9.1 |
|  | H-5 | 9.53(1H,d) | 9.0 |
|  | H-5’ | 9.57(1H,d) | 9.5 |
| ^13^C-NMR | C-2 | 159.44 |  |
|  | C-2’ | 159.38 |  |
|  | C-7’ | 156.78 |  |
|  | C-7 | 154.47 |  |
|  | C-4,4’ | 154.27 |  |
|  | C-10a’ | 134.46 |  |
|  | C-10a | 134.22 |  |
|  | C-8a’ | 133.26 |  |
|  | C-8a | 133.04 |  |
|  | C-5 | 129.36 |  |
|  | C-5’ | 129.22 |  |
|  | C-9’ | 127.75 |  |
|  | C-9 | 127.48 |  |
|  | C-5a’ | 125.20 |  |
|  | C-10’ | 124.86 |  |
|  | C-10 | 124.66 |  |
|  | C-5a | 124.49 |  |
|  | C-6 | 116.60 |  |
|  | C-6’ | 116.38 |  |
|  | C-4a’ | 115.70 |  |
|  | C-4a | 115.54 |  |
|  | C-8 | 111.11 |  |
|  | C-1’ | 109.19 |  |
|  | C-1 | 109.09 |  |
|  | C-8’ | 108.26 |  |
|  | C-3’ | 99.54 |  |
|  | C-3 | 99.53 |  |
|  | 2’-OCH_3_ | 55.13 |  |
|  | 2-OCH_3_ | 55.10 |  |
|  | 7’-OCH_3_ | 54.59 |  |

Compound 8

|  | Position | δ (ppm) | J (Hz) |
| --- | --- | --- | --- |
| ^1^H-NMR | 8’-OCH_3_ | 3.85 (3H,s) |  |
|  | 2-OCH_3_ | 4.20 (3H,s) |  |
|  | 2’-OCH_3_ | 4.21 (3H,s) |  |
|  | H-3’ | 7.04(1H,s) |  |
|  | H-3 | 7.05(1H,s) |  |
|  | H-10’ | 7.06(1H,d) | 9.4 |
|  | H-10 | 7.12(1H,d) | 9.4 |
|  | H-8’ | 7.20(1H,d) | 2.8 |
|  | H-6’ | 7.22(1H,dd) | 9.4,2.8 |
|  | H-6 | 7.27(1H,d) | 9.4 |
|  | H-9’ | 7.40(1H,d) | 9.2 |
|  | H-9 | 7.79(1H,d) | 9.4 |
|  | H-5 | 9.34(1H,d) | 9.4 |
|  | H-5’ | 9.53(1H,d) | 8.9 |
| ^13^C-NMR | C-2’ | 159.52 |  |
|  | C-2 | 159.389 |  |
|  | C-7’ | 154.46 |  |
|  | C-4,4’ | 154.28 |  |
|  | C-7 | 145.79 |  |
|  | C-8 | 141.15 |  |
|  | C-8a’ | 134.24 |  |
|  | C-8a | 134.16 |  |
|  | C-10a’ | 133.27 |  |
|  | C-5’ | 129.38 |  |
|  | C-9’ | 127.51 |  |
|  | C-10a | 126.71 |  |
|  | C-5a,5a’ | 125.36 |  |
|  | C-10,10’ | 124.69 |  |
|  | C-5 | 124.50 |  |
|  | C-9 | 120.73 |  |
|  | C-6 | 117.09 |  |
|  | C-6’ | 116.61 |  |
|  | C-4a’ | 115.78 |  |
|  | C-4a | 115.72 |  |
|  | C-8’ | 111.13 |  |
|  | C-1 | 109.20 |  |
|  | C-1’ | 109.12 |  |
|  | C-3 | 99.65 |  |
|  | C-3’ | 99.54 |  |
|  | 8-OCH_3_ | 60.50 |  |
|  | 2’-OCH_3_ | 55.15 |  |
|  | 2-OCH_3_ | 55.12 |  |

Compound 9

|  | Position | δ (ppm) | J (Hz) |
| --- | --- | --- | --- |
| ^1^H-NMR | H-9,10 | 2.64(4H,s) |  |
|  | 2-OCH_3_ | 3.74(3H,s) |  |
|  | H-3 | 6.42(1H,d) | 2.6 |
|  | H-1 | 6.49(1H,d) | 2.6 |
|  | H-8 | 6.82(1H,d) | 7.2,0.84 |
|  | H-6 | 6.84(1H,dd) | 8.1,0.84 |
|  | H-7 | 7.07(1H,m) |  |
| ^13^C-NMR | C-2 | 160.43 |  |
|  | C-4 | 155.60 |  |
|  | C-5 | 153.45 |  |
|  | C-10a | 143.40 |  |
|  | C-8a | 141.68 |  |
|  | C-7 | 128.40 |  |
|  | C-4a | 122.15 |  |
|  | C-5a | 120.84 |  |
|  | C-8 | 117.45 |  |
|  | C-6 | 115.01 |  |
|  | C-1 | 107.40 |  |
|  | C-3 | 102.68 |  |
|  | 2-OCH_3_ | 56.31 |  |
|  | C-9 | 32.12 |  |
|  | C-10 | 31.63 |  |

Compound 10

|  | Position | δ (ppm) | J (Hz) |
| --- | --- | --- | --- |
| ^1^H-NMR | 7-OCH_3_ | 3.88(3H,s) |  |
|  | 4-OCH_3_ | 4.04(3H,s) |  |
|  | H-3 | 6.79(1H,d) | 2.6 |
|  | H-1 | 6.86(1H,d) | 2.6 |
|  | H-6 | 7.19(1H,d) | 8.6,2.3 |
|  | H-8 | 7.37(1H,d) | 2.3 |
|  | H-9 | 7.58(1H,d) | 8.5 |
|  | H-10 | 7.67(1H,d) | 8.5 |
|  | H-5 | 9.32(1H,d) | 9.1 |
|  | 2-OH | 9.82(1H,s) |  |
| ^13^C-NMR | C-4 | 160.18 |  |
|  | C-7 | 157.37 |  |
|  | C-2 | 156.88 |  |
|  | C-8a | 135.49 |  |
|  | C-10a | 133.95 |  |
|  | C-5 | 129.83 |  |
|  | C-9 | 128.84 |  |
|  | C-10 | 128.45 |  |
|  | C-5a | 125.65 |  |
|  | C-6 | 117.56 |  |
|  | C-4a | 114.85 |  |
|  | C-8 | 110.14 |  |
|  | C-1 | 105.90 |  |
|  | C-3 | 101.14 |  |
|  | 4-OCH_3_ | 56.98 |  |
|  | 7-OCH_3_ | 56.38 |  |

Compound 11

|  | Position | δ (ppm) | J (Hz) |
| --- | --- | --- | --- |
| ^1^H-NMR | H-10’ | 2.30(2H,m) |  |
|  | H-9’ | 2.46(2H,m) |  |
|  | 4’-OCH_3_ | 3.85 (3H,s) |  |
|  | 7-OCH_3_ | 3.88 (3H,s) |  |
|  | 7’-OCH_3_ | 3.96 (3H,s) |  |
|  | 4-OCH_3_ | 4.09 (3H,s) |  |
|  | H-8’ | 6.12(1H,s) |  |
|  | H-1’ | 6.26(1H,d) | 1.9 |
|  | H-3’ | 6.40(1H,d) | 2.1 |
|  | H-3 | 7.01(1H,s) |  |
|  | H-6 | 7.25(1H,dd) | 9.5,2.5 |
|  | H-8 | 7.37(1H,d) | 2.8 |
|  | H-10 | 7.65(1H,d) | 9.1 |
|  | H-9 | 7.71(1H,d) | 9.2 |
|  | H-5’ | 7.93(1H,s) |  |
|  | H-5 | 9.40(1H,d) | 9.5 |
|  | 2’-OH | 9.50(1H,s) |  |
|  | 7-OH | 9.86(1H,s) |  |
| ^13^C-NMR | C-4’ | 158.72 |  |
|  | C-2’ | 158.22 |  |
|  | C-7 | 157.72 |  |
|  | C-4 | 156.71 |  |
|  | C-2 | 148.41 |  |
|  | C-7’ | 147.30 |  |
|  | C-6’ | 147.07 |  |
|  | C-10a’ | 141.53 |  |
|  | C-8a’ | 133.86 |  |
|  | C-10a | 130.8 |  |
|  | C-8a | 130.18 |  |
|  | C-5 | 130.05 |  |
|  | C-9 | 129.56 |  |
|  | C-5a’ | 128.76 |  |
|  | C-5a | 127.55 |  |
|  | C-4a’ | 125.57 |  |
|  | C-10 | 121.31 |  |
|  | C-6 | 118.10 |  |
|  | C-4a | 115.29 |  |
|  | C-1 | 115.26 |  |
|  | C-5’ | 114.25 |  |
|  | C-8’ | 113.77 |  |
|  | C-8 | 110.12 |  |
|  | C-1’ | 108.81 |  |
|  | C-3 | 101.81 |  |
|  | C-6’ | 99.60 |  |
|  | 6’-OCH_3_ | 57.30 |  |
|  | 4-OCH_3_ | 57.14 |  |
|  | 4’-OCH_3_ | 56.79 |  |
|  | 7-OCH_3_ | 56.43 |  |
|  | C-9’ | 31.46 |  |
|  | C-10’ | 29.73 |  |

Compound 12

|  | Position | δ (ppm) | J (Hz) |
| --- | --- | --- | --- |
| ^1^H-NMR | 4-OCH_3_ | 4.05(3H,s) |  |
|  | H-3 | 6.82(1H,d) | 2.6 |
|  | H-1 | 6.90(1H,d) | 2.6 |
|  | H-6 | 7.17(1H,d) | 8.6,2.3 |
|  | H-8 | 7.23(1H,d) | 2.3 |
|  | H-10 | 7.54(1H,d) | 8.5 |
|  | H-9 | 7.57(1H,d) | 8.5 |
|  | H-5 | 9.32(1H,d) | 9.3 |
|  | 2,7-OH | 9.70(2H,s) |  |
| ^13^C-NMR | C-4 | 160.12 |  |
|  | C-2 | 156.57 |  |
|  | C-7 | 155.63 |  |
|  | C-8a | 135.26 |  |
|  | C-10a | 134.25 |  |
|  | C-5 | 129.99 |  |
|  | C-9 | 128.66 |  |
|  | C-10 | 128.23 |  |
|  | C-5a | 124.70 |  |
|  | C-6 | 118.11 |  |
|  | C-4a | 115.17 |  |
|  | C-8 | 112.79 |  |
|  | C-1 | 106.00 |  |
|  | C-3 | 101.07 |  |
|  | 4-OCH_3_ | 56.92 |  |
